# Octanoylation of early intermediates of mycobacterial methylglucose lipopolysaccharides

**DOI:** 10.1038/srep13610

**Published:** 2015-09-01

**Authors:** Ana Maranha, Patrick J. Moynihan, Vanessa Miranda, Eva Correia Lourenço, Daniela Nunes-Costa, Joana S. Fraga, Pedro José Barbosa Pereira, Sandra Macedo-Ribeiro, M. Rita Ventura, Anthony J. Clarke, Nuno Empadinhas

**Affiliations:** 1 CNC – Center for Neuroscience and Cell Biology, University of Coimbra, 3004-517 Coimbra, Portugal; 2 Department of Molecular and Cellular Biology, University of Guelph, Ontario, Canada; 3 ITQB – Instituto de Tecnologia Química Biológica, Universidade Nova de Lisboa, Portugal; 4 IBMC – Instituto de Biologia Molecular e Celular, Universidade do Porto, Portugal; 5 Instituto de Investigação e Inovação em Saúde, Universidade do Porto, Portugal; 6 III/UC– Instituto de Investigação Interdisciplinar, University of Coimbra, Portugal

## Abstract

Mycobacteria synthesize unique intracellular methylglucose lipopolysaccharides (MGLP) proposed to modulate fatty acid metabolism. In addition to the partial esterification of glucose or methylglucose units with short-chain fatty acids, octanoate was invariably detected on the MGLP reducing end. We have identified a novel sugar octanoyltransferase (OctT) that efficiently transfers octanoate to glucosylglycerate (GG) and diglucosylglycerate (DGG), the earliest intermediates in MGLP biosynthesis. Enzymatic studies, synthetic chemistry, NMR spectroscopy and mass spectrometry approaches suggest that, in contrast to the prevailing consensus, octanoate is not esterified to the primary hydroxyl group of glycerate but instead to the C6 OH of the second glucose in DGG. These observations raise important new questions about the MGLP reducing end architecture and about subsequent biosynthetic steps. Functional characterization of this unique octanoyltransferase, whose gene has been proposed to be essential for *M. tuberculosis* growth, adds new insights into a vital mycobacterial pathway, which may inspire new drug discovery strategies.

The genus *Mycobacterium* includes several successful pathogens causing hard-to-eradicate diseases, such as leprosy and tuberculosis (TB). Although the number of people infected with drug-sensitive TB is declining each year, serious hurdles in controlling the disease are imposed by new emerging multidrug-resistant strains and growing incidence of debilitating chronic diseases and other immune vulnerabilities^1^,^2^.

Mycobacterial pathogenesis is intimately associated to a unique lipid-rich cell wall^3^, to several distinctive metabolic pathways and to rare macromolecules that include intracellular α–(1→4)-linked polymethylated polysaccharides (PMPSs) of 3–*O*–methylmannose (MMP) and of 6–*O*–methylglucose (MGLP)^4^,^5^,^6^. Although experimental evidence for the biological functions of PMPSs *in vivo* is still missing, they were proven to form stable 1:1 complexes with long-chain fatty acids *in vitro* modulating their synthesis through interaction with fatty acid synthase I (FAS-I)^7^,^8^. Due to α–(1→4)-linked backbones, PMPSs likely assume a helical conformation in solution with inward-facing methyl groups forming a hydrophobic cavity capable of accommodating newly synthesized acyl-CoA chains^9^. This interaction was proposed to: (i) terminate the elongation and promote diffusion of neo-synthesized acyl chains relieving FAS-I product inhibition^7^,^10^; (ii) protect acyl-CoAs from degradation by cytoplasmic lipolytic enzymes; (iii) prevent disruption of metabolism likely resulting from cytoplasmic accumulation of fatty acids^8^. PMPSs raise the overall FAs synthetic rate and modulate the length of the newly synthesized fatty acyl-CoAs^11^,^12^ and seem to be essential for adaptation to thermal stress, although the precise mechanism remains unclear^4^,^13^. While MMP is apparently restricted to rapidly growing mycobacteria, MGLP has been detected in all *Mycobacterium* spp. examined thus far and is the only type of PMPS in the pathogen *Mycobacterium tuberculosis*^4^,^6^.

MGLP is composed of 15–20 α–(1→4)–linked glucose or 6–*O*–methylglucose units with a 3-*O*-methylglucose non-reducing end^6^. In the mature MGLP structure, short-chain fatty acids (namely acetate, propionate or isobutyrate) are often esterified to the glucose units near the non-reducing end^14^,^15^. The polysaccharide’s reducing end is composed of diglucosylglycerate (α–glucosyl–(1→6)–α–glucosyl–(1→2)–glycerate; DGG) and the second glucose of DGG is α–(1→4)-linked to the MGLP main chain, which contains two additional branching β–(1→3)-linked glucoses to which succinate may be esterified^4^,^16^. An octanoate moiety was initially detected at the terminal glycerate and suggested to act as the anchor of MGLP intermediates to the cytoplasmic membrane during the elongation steps^14^. Octanoate has also been proposed to raise the interaction energy required for complexation with longer acyl-CoAs, which might confer further discriminatory ability to MGLP^17^. In addition, octanoate was suggested to be the axis around which MGLP could fold in the absence of longer acyl-CoAs, contributing to stabilizing its helical conformation^17^. In *M. phlei*, MGLP acylation levels were shown to regulate methylation, which could only be observed after partial acetylation of glucoligosaccharide acceptors^18^. This could explain octanoylation as the basal acylation required for 6–O methylation to occur.

Although MGLP was identified in the 1960s^19^ and its chemical composition, structure and interactions with fatty acids have been thoroughly examined, its biosynthetic pathway remained largely unknown^4^. Glucosylglycerate (GG), the putative primer for MGLP synthesis, is now known to arise from the consecutive action of the enzymes glucosyl-3-phosphoglycerate synthase (GpgS) and glucosyl-3-phosphoglycerate phosphatase (GpgP), encoded in *M. tuberculosis* H37Rv by *Rv1208* and *Rv2419c*, respectively^20^,^21^,^22^,^23^. A GG hydrolase was recently identified in rapidly-growing mycobacteria but its involvement in the regulation of MGLP biosynthesis has not been examined^24^. The third MGLP biosynthetic step was predicted to involve the transfer of an additional glucose to GG to yield DGG^13^. Given that bacterial genes involved in one pathway are often grouped into operons^25^, confirmation of the genetic implication of Rv3032 (α–(1→4)-glycosyltransferase) and Rv3030 (6–*O*–methyltransferase) in MGLP extension and methylation, respectively, linked Rv3031 (a putative glycoside hydrolase similar to α–(1→6)-branching enzymes) to DGG synthesis^6^,^13^ (Fig. 1). After assigning GpgP activity to Rv2419c we deemed it important to investigate the function of the neighboring gene considered essential for *M. tuberculosis* H37Rv growth^23^,^26^,^27^. In addition to *Rv2418c,* we cloned the *M. smegmatis* ortholog and that from the thermophilic *M. hassiacum*, for protein stability constraints often curbing functional and structural studies^28^.

Herein, we show that Rv2418c catalyzes the transfer of octanoate and to a lesser extent, some short-chain fatty acids, to DGG and GG *in vitro*. Surprisingly, we found that octanoate is not transferred to the glycerate moiety as previously reported but instead to the C6 OH of the second glucose in DGG. This was confirmed by comparison of the enzymatic product with both chemically synthesized versions of octanoylated DGG. The implication of this novel octanoyltransferase (OctT) in the biosynthetic pathway to MGLP furthers our knowledge on the molecular glycobiology of mycobacteria and offers additional tools for drug discovery.

## Results

### Identification, sequence analyses, recombinant expression and purification of OctT

**Figure 1:**
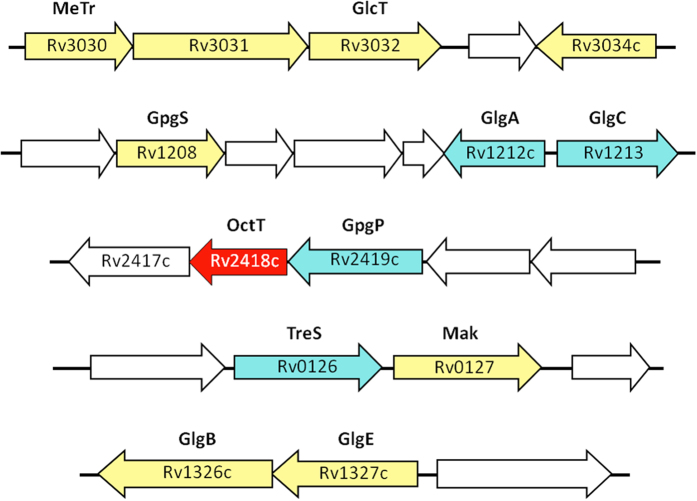
*M. tuberculosis* H37Rv genomic clusters proposed to participate in MGLP biosynthesis. *Yellow* and *red*, genes proposed to be essential for *M. tuberculosis* H37Rv growth by saturation transposon mutagenesis^27^. *Blue*, genes with confirmed function but considered non-essential for growth. *White*, genes with unknown or putative function that lack experimental confirmation. MeTr, probable methyltransferase; GlcT; α(1→4)-glycosyltransferase; GpgS, glucosyl-3-phosphoglycerate synthase; GlgA, glycogen synthase; GlgC, glucose-1-phosphate adenylyltransferase; OctT, DGG-octanoyltransferase (Sequence data are shown in Figure S1); GpgP, glucosyl-3-phosphoglycerate phosphatase; TreS, trehalose synthase; Mak, maltokinase; GlgB, glycogen-branching enzyme; GlgE, maltosyltransferase.

The genes adjacent to the recently identified mycobacterial glucosyl-3-phosphoglycerate phosphatase (GpgP, *Rv2419c*)^23^ encoded several enzymes of unknown function as well as the versatile acetyltransferase Rv2416c^29^ (Fig. 1). *Rv2417c*, one of the genes with unknown function in this region, showed 24% amino acid sequence identity with TM841, a *Thermotoga maritima* protein of unknown function belonging to the DEGV family^30^. The crystal structure of TM841 has been determined (PDB entry: 1MGP) and a fatty acid molecule was identified at a non-conserved interface between the two α/β domains, suggesting a role as fatty acid carrier protein or in fatty acid metabolism^30^. The contiguous *Rv2418c* was annotated as a possible lysophospholipase belonging to the SGNH_hydrolase (or GDSL_hydrolase, PF00657) superfamily, whose members contain a conserved GDSL motif characteristic of lipases, acylhydrolases and esterases^31^. BLAST analyses with the Rv2418c amino acid sequence (OctT) revealed hypothetical homologs in most sequenced mycobacterial genomes (79–100% amino acid identity). Strains of the species *M. abscessus* had the most divergent sequences among the mycobacteria analyzed (71% identity) and, so far, only *M. leprae* lacked a detectable OctT homolog. Members of closely related genera of the suborder Corynebacterineae such as *Rhodococcus*, *Nocardia* and others, but not of the genus *Corynebacterium*, also possess hypothetical OctT orthologs with significant amino acid identity (>50%). Among these, only a few strains of *Nocardia* are known to synthesize MGLP^32^. OctT orthologs have also been detected in strains of *Streptomyces* (WP_009081052). However, these strains’ taxonomical classification has not been clearly defined. The closely related *Streptomyces griseus* has been reported to produce a form of acetylated methylmannose polysaccharide (MMP) although limited details about its structure are available^33^. An alignment (Fig. S1) of selected putative actinobacterial OctTs shows that, despite the annotation as SGNH/GDSL esterases, they do possess the four characteristic and invariant Ser, Gly, Asn, and His residues and the postulated Ser-His-Asp catalytic triad required for activity, but not the highly conserved and distinct GDSL region^31^, which is here limited to DSL. The peptidoglycan *O*-Acetyltransferase B (PatB), initially annotated as a GDSL hydrolase (with the catalytic triad of serine esterases), was proven to be a bona-fide acyltransferase with only weak esterase activity^34^. Site-directed mutagenesis on PatB confirmed the identity of the catalytic residues^35^.

No *octT* orthologs could be detected in bacterial genomes outside the class Actinobacteria. The *M. tuberculosis*, *M. smegmatis* and *M. hassiacum* homologs were cloned and expressed in *E. coli* in order to functionally characterize their encoded polypeptides. Purification of soluble and bioactive recombinant OctTs required including β-mercapthoethanol (7.5 mM) and Tween 20 (0.1% vol/vol) in all the buffers throughout the purification procedure. Recombinant OctT production was confirmed by Western immunoblot analysis and peptide mass fingerprinting (IPATIMUP Proteomics Unit), which indicated the presence of two forms of His-tagged OctT, one with the expected mass and another with much higher mass (Fig. S2). Gel filtration experiments indicated that recombinant His-tagged OctTs could be separated into two populations: a subpopulation of molecules with a molecular mass of 111.1 ± 6.9 kDa (*M. tuberculosis*), 126.6 ± 6.9 kDa (*M. smegmatis*) or 125.2 ± 6.9 kDa (*M. hassiacum*), compatible with tetrameric architectures, and another fraction corresponding to a higher order oligomer, later shown to be inactive (see below). The three recombinant enzymes were purified to apparent homogeneity as determined by SDS-PAGE analysis (Fig. S2) with yields of 1.2 mg (*M. tuberculosis*), 10 mg (*M. smegmatis*) or 3 mg (*M. hassiacum*) of OctT per liter of culture.

### Properties of recombinant OctT

The recombinant OctT from *M. hassiacum*, the most thermophilic species among known mycobacteria, was selected for characterization given its expectably higher stability^28^. The *M. hassiacum* OctT was very selective towards the acceptor sugars and from the array of compounds tested it could only efficiently use GG and DGG (Table 1A), with a detectable preference toward DGG (Table 2). We could only measure a significant transfer of ester groups from CoA- or *p*NP-activated octanoate and hexanoate while vestigial transfer of other acyl-CoAs could only be detected by mass spectrometry (described below) (Table 1A). For convenience, the synthetic *p*NP esters were used to characterize *M. hassiacum* OctT temperature profile, pH dependence and the effect of divalent cations while the kinetic parameters were examined with CoA-activated substrates hexanoyl-CoA (Hex-CoA) and octanoyl-CoA (Oct-CoA) as donors. However, because the kinetic constants for the *M. hassiacum* OctT determined at 37 °C were very low (Table 2) it was deemed essential to assess if this was a characteristic of this particular enzyme or a property shared by other OctTs. Thus, relevant kinetic parameters were also calculated for the OctT from *M. smegmatis* (optimal growth at 37 °C), which revealed much higher turnover and catalytic efficiency (Table 2). Both recombinant OctTs exhibited Michaelis-Menten kinetic behavior at 37 °C with Oct-CoA or Hex-CoA up to a concentration of 100 μM (Fig. S4). Higher concentrations of these ester donors in mixtures containing 20 mM or 45 mM GG or DGG were progressively inhibitory of enzyme activity (Fig. S5). A possible inhibitory effect of free CoA, free octanoate or Oct-DGG product on enzyme activity was tested. Free CoA and free octanoate showed no effect on the reaction rate or amount of product formed while the addition of DGG-Oct was slightly inhibitory with 22% loss of activity in the presence of 400 μM of added product (Fig S5).

Despite being native to the moderate thermophile *M. hassiacum* with optimal growth around 50 °C^28^, the optimal temperature for this OctT activity *in vitro* ranged from 37 to 45 °C with maximal activity at 45 °C and sharply decreasing above this temperature (Fig. S3A). At 37 °C the enzyme was maximally active between pH 7.0 and 8.5 (Fig. S3B) and in the absence of added cations although EDTA slightly inhibited the activity (27% decrease), similar to the effect observed for most cations at 5 mM (Zn^2+^ > Mg^2+^ > Co^2+^; Fig. S3C).

The OctTs studied did not exhibit detectable esterase activity towards Oct-DGG under the conditions tested (see Methods).

### Synthesis of (2*R*)-2-*O*-[6-*O*-octanoyl-(α-d-glucopyranosyl-(1→6)-α-d-glucopyranosyl]-2,3-dihydroxypropanoic acid 1 and of (2*R*)-2-*O*-(α-d-glucopyranosyl-(1→6)-α- d-glucopyranosyl)-3-*O*-octanoyl-2,3-dihydroxypropanoic acid 2

The enzymatic synthesis of Oct-DGG was carried out at supra optimal concentrations of acyl donor (1.5 mM) in order to obtain substantial amounts of product for NMR data acquisition. Efforts to purify the related OctT product octanoyl-GG were unsuccessful as only trace amounts could be obtained. When using GG at higher acyl donor concentrations, the enzyme was inhibited and yielded insignificant product levels (Fig. S5).

**Figure 2:**
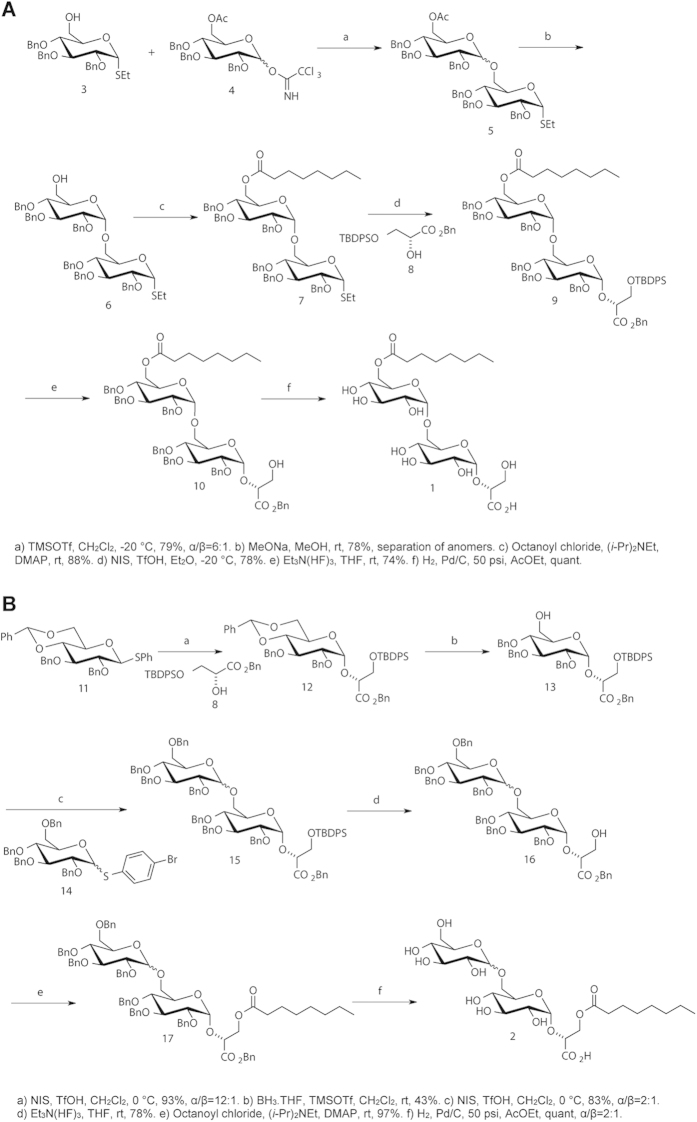
(**A**) Synthesis of (2*R*)-2-*O*-[6-*O*-octanoyl-(α-d-glucopyranosyl-(1→6)-α-d-glucopyranosyl]-2,3-dihydroxypropanoic acid **1** (6-Oct-DGG). (**B**) Synthesis of (2*R*)-2-*O*-(α-d-glucopyranosyl-(1→6)-α-d-glucopyranosyl)-3-*O*-octanoyl-2,3-dihydroxypropanoic acid **2** (DGG-3-Oct).

The synthetic route for 6-Oct-DGG **1** (enzymatically obtained product) and for DGG-3-Oct **2** (mimicking the reported MGLP reducing end) are illustrated in Fig. 2. These syntheses presented several challenges and our previous synthetic strategy of the related natural DGG [(2*R*)-2-*O*-α-d-glucopyranosyl-(1→6)-α-d-glucopyranosyl-2,3-dihydroxypropanoate] required extensive modifications^36^. An additional ester, the 6-octanoate, needed to be introduced, and consequently the protecting group for the carboxylic acid of the glycerate moiety could not be a methyl ester as used previously^36^,^37^. Thus, the benzyl glycerate derivative **8** (benzyl (2R)-3-*O*-*tert*-butyldiphenylsilyl-2,3-dihydroxypropanoate; see Supplemental Information) was synthesized and used in the synthesis of **1** and **2**, and the benzyl ester was easily removed in the final step by hydrogenation (Fig. 2), leaving the octanoate group undisturbed. The free acids of **1** and **2** were obtained instead of the corresponding inorganic salts.

**Figure 3:**
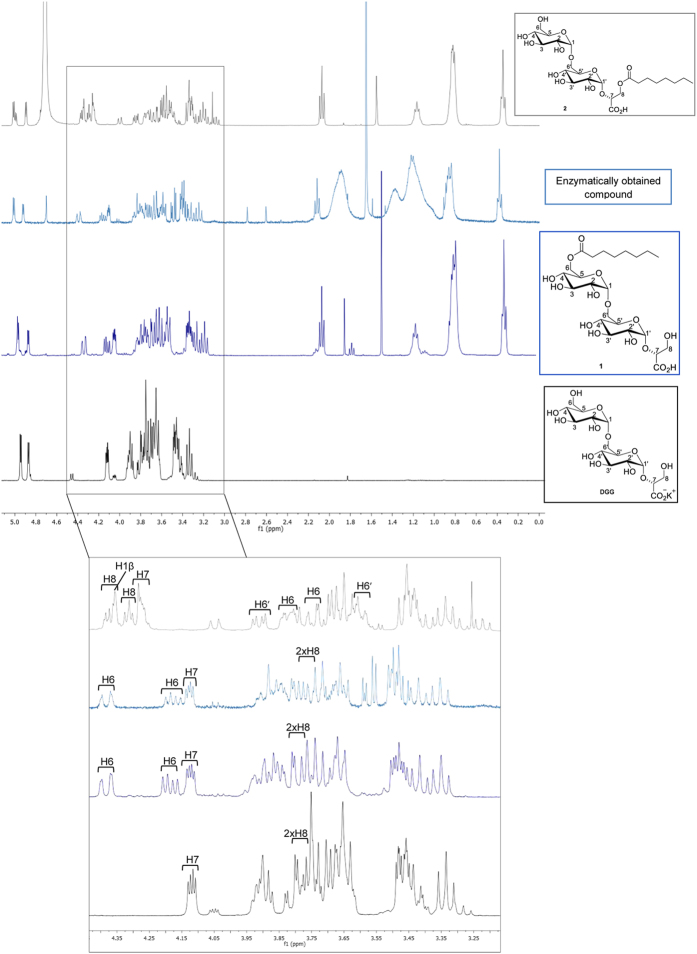
^1^H-NMR spectra of natural Oct-DGG, chemical synthesized compounds 1 and 2 and DGG. Peak assignment was supported by 2D correlation NMR experiments (COSY and HMQC).

The glycosylation reaction between trichloroacetimidate **4**^38^,^39^ and acceptor **3**^40^ using TMSOTf as the promoter, afforded the disaccharide **5**, with 79% yield and an α/β ratio of 6:1 (Fig. 3A). After deprotection of the 6-OAc group, it was possible to separate the anomers by column chromatography. Proceeding with the α anomer, octanoylation of the free primary hydroxyl group afforded product **7** in excellent yield (88%). The second glycosylation reaction, using glycerate **8** as the acceptor and employing NIS/TfOH as the activating system, was highly stereoselective and afforded exclusively the corresponding α glucoside **9** in 78%. After selective fluorolysis of the silyl ether with triethylamine trihydrofluoride followed by hydrogenation to remove the benzyl groups the final compound **1** was efficiently obtained. The overall yield for the synthesis of 6-Oct-DGG **1** starting from the thioglucoside **3** was 27%.

It has been previously described that the octanoyl group of the MGLP esterified the primary alcohol of the glycerate moeity of the polysaccharide reducing end^14^. In order to confirm the position of the octanoate in the MGLP reducing end structure, we also developed the synthesis of DGG-3-Oct **2**, as described in (Fig. 2B). Benzyl glycerate **8** was the acceptor in the first glycosylation reaction with the thioglucoside **11**^36^,^41^, in the presence of NIS/TfOH, and the glucoside **12** was obtained in an excellent α/β ratio (12:1) and yield (93%). The regioselective ring opening of the 4,6-benzylidene acetal with borane tetrahydrofurane complex and TMSOTf^42^ afforded the corresponding 4-benzyl ether **13** in 43% yield. The major byproduct was the diol. Alcohol **13** was the acceptor in the next glycosylation reaction with thioglucoside donor **14**^43^. The anomeric selectivity was only α/β = 2:1, as it was expected since no electronegative ester group was present at the C-6 position of the donor^44^. However, the presence of an ester at this position was not possible, because its cleavage to afford the final product would also cleave the octanoate. Desylilation of the glycerate alcohol and reaction with octanoyl chloride, diisopropylethylamine and DMAP in dichloromethane afforded **17**, the yields being 78% and 97% for the consecutive reactions, respectively. Hydrogenation to remove the benzyl groups afforded the final product **2**, in quantitative yield, as a 2:1 mixture of the α- and β-anomers.

It can be concluded that the proton NMR spectrum of the enzymatically obtained compound is identical to that of compound **1** (due to its purification process, the NMR spectrum of the enzymatic Oct-DGG has some impurities that appear as extra peaks) (Fig. 3). We have also compared these NMR spectra to that obtained for the enzyme substrate DGG^36^. In DGG and compound **1**, the easily identifiable H7 chemical shift is very similar, whereas in compound **2** this signal appears downfield, indicating that an electron withdrawing group, such as the octanoate ester, is esterifying the vicinal hydroxyl group. Furthermore, the CH_2_ group of the glycerate (two H8 signals – AB system) has shifted considerably downfield, confirming this assumption. With compound **1**, and because the octanoate is esterifying the free C-6 hydroxyl group, these signals (two H6 signals – AB system) are significantly shifted downfield. The chemical synthesis of both compounds **1** and **2** was a fundamental resource to unequivocally confirm the structure of the natural Oct-DGG.

### Mass spectrometry

**Figure 4:**
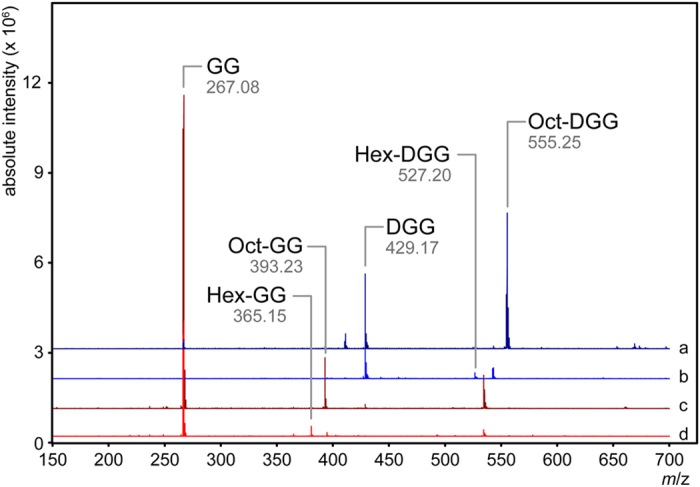
Mass spectra of OctT reaction products. DGG (blue spectra) and GG (red spectra) were incubated in the presence and absence of OctT from *M. hassiacum* with either Oct-CoA (a/c) or Hex-CoA (b/d). Unique peaks not present in control reactions are identified. Spectra were analyzed using the open-source mMass software package^74^ and all identified masses are the [M-H]^-1^ ions.

**Figure 5:**
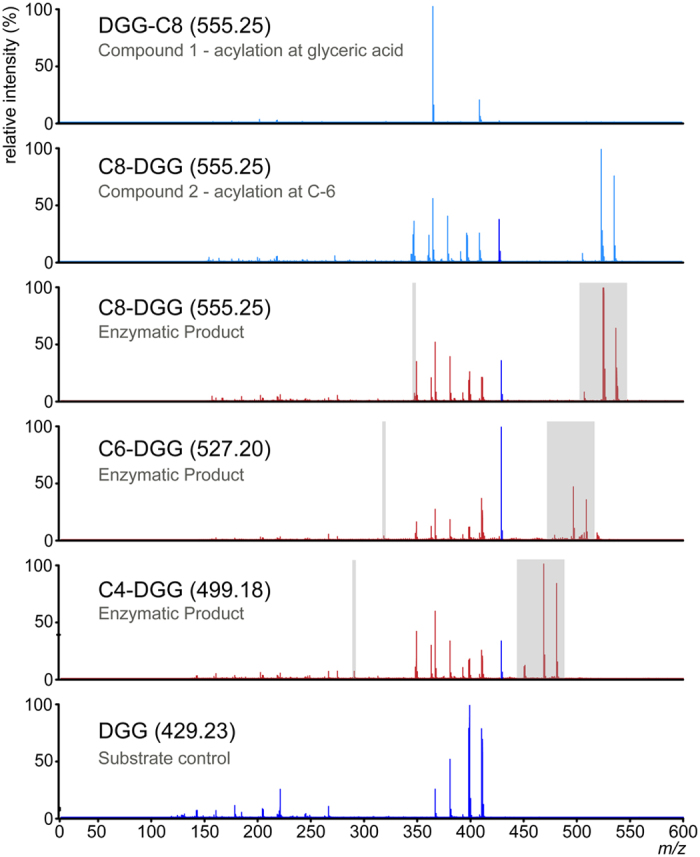
MS/MS analysis of DGG and of its modified variants. In order to validate the site of modification on the OctT reaction products MS/MS spectra were collected for the NMR-validated material (compounds 1 and 2; light blue), unmodified DGG (dark blue) and the C4, C6 and C8 OctT reaction products (red). A limited number of ions were observed which are unique to each modified reaction product and are shifted by 28 Da with the corresponding reduction in acyl-donor length (highlighted grey). Spectra were normalized and analyzed using the open-source mMass software^74^. Parental ions are indicated in parenthesis and are the [M-H]^−1^ ions.

To overcome the constraints inherent to monitoring product formation using a colorimetric reporter (either *p*NP or DTNB) and to the limited sensitivity of enzymatic quantification with several of the acyl activated donors, direct observation of the product was sought using mass spectrometry (Fig. 4). We found that product formation is consistent with those reactions where a rate of *p*NP or CoA release was quantifiable and for which enzyme kinetics could be analyzed (Table 2). Confirmation of the site of acyl modification was initially sought using tandem MS of enzymatically derived products. Unfortunately, relatively few peaks were observed that are unique to modified products and therefore a reconstruction of the product was not possible. With this in mind an NMR analysis was used to interrogate the major enzymatically derived product, which indicated it was consistent with compound **1**, Oct-DGG, with the acyl modification on the C6 OH of the second glucose. This was further confirmed by comparing the MS/MS fragmentation of the enzymatically derived material with the fragmentation of the synthetic material (Fig. 5).

**Figure 6:**
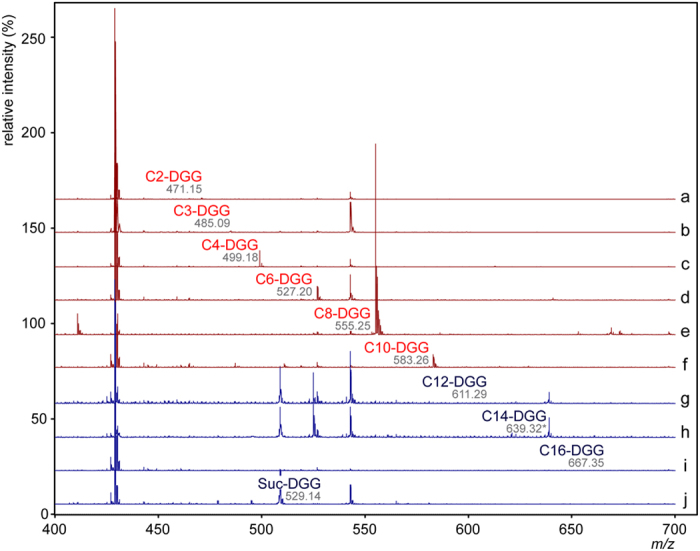
OctT acyl-donor promiscuity. DGG was incubated individually with acyl-CoAs including C2 (a), C3 (b), C4 (c), C6 (d), C8 (e), C10 (f), C12 (g) C14 (h) and C16 (i) in addition to succinyl-CoA (j) in the presence of OctT from *M. hassiacum*. Mass spectra from reactions where transferase activity was observed are colored red while spectra lacking product are colored blue. In all cases except for C14 the spectra from control reactions lacked the observed product peaks. A peak at the expected size of the C14 reaction product was observed in both control reactions and the C12 spectra. Its presence in those spectra and the lack of product in the C12 and C16 reactions makes it unlikely that this ion represents an authentic DGG-C14 reaction product. Spectra were normalized and analyzed using the open-source mMass software package^74^ and all identified masses are the [M-H]^−1^ ions.

Product could be detected with donors from acetyl-CoA (C2) to decanoyl-CoA (C10) but absent when donors with an acyl chain longer than C10 were used (Table 1A) (Fig. 6). Relative ionization is consistent with the enzymatic rates observed using the kinetic assay, however quantitation of reaction products by MS was not possible without suitable standards. The acidic acyl substrate succinyl-CoA was also tested and product formation could not be detected with any acceptor utilized (Table 1A–C). Once a reconstruction of Oct-DGG product was possible by comparison to NMR analyses, we examined the site of modification for the most relevant acyl donors with DGG as acceptor and concluded that regardless of acyl chain size the modification is invariably present on the second glucose C6 OH position (Fig. 5).

Previous studies anticipated that octanoylation position should occur in the primary hydroxyl group of the glyceric acid^14^,^15^. After assigning the acylation position to the second glucose of DGG we wondered about the importance of glyceric acid (GA) in the acyl transfer reaction. A combination of compounds mimicking the preferred substrates and analogs were tested, including the combination isomaltose + GA mimicking DGG or glucose + GA mimicking GG. The disaccharides trehalose, maltose and isomaltose, isolated or in combination with glyceric acid, were substrates for the enzyme but no discernible difference was detected in the presence or absence of GA (Table 1B). Although product was detected with these disaccharides the reaction rates were extremely low and below the detection limit of the enzymatic assay. In order to test donor specificity an array of substrates were analyzed as shown in Table 1B. Substrates analogous to the preferred acceptors [DGG (α–d–glucosyl–(1→6)–α–d–glucosyl–(1→2)–d–glycerate) and GG (α–d–glucosyl–(1→2)– d–glycerate)] were examined and product formation was detected with MGG (α–d–mannopyranosyl–(1→2)–α–d–glucopyranosyl–(1→2)–d-glycerate) but not MG (α-d-mannosyl-(1→2)-d-glycerate) (Table 1B). Although we cannot exclude MGG as a genuine substrate this vestigial utilization is most likely due to trace amounts of GG in the MGG sample in result of degradation and not to real mannosyl substrate usage. MGG is an extremely rare compatible solute that so far has been identified in very few organisms, none of which Actinobacteria^45^,^46^.

The proposed involvement of OctT in the pathway for mature MGLP (polyacylated) led us to also test several possible acceptor substrates mimicking distinct parts of the polysaccharide that are often acylated with short-chain fatty acids^4^. Maltooligosaccharides mimicking the MGLP non reducing end (maltotriose, maltotetraose, maltopentaose, maltoheptaose) were tested as acceptors in combination with the preferred octanoyl substrate as well as with activated acetyl and butyryl typically found in this MGLP region, but no product could be detected for any of the reactions (Table 1C). The disaccharide laminaribiose (Lam) mimicking the β-(1→3) branching units attached to the MGLP main chain was also tested to but no product could be detected with either Oct-CoA or Suc-CoA (succinyl is commonly found in these branching units) as donors (Table 1C). These results strongly indicate that the purified OctT can only efficiently use a very narrow range of glucosyl acceptors *in vitro*, which strengthens the hypothesis of a role in the specific acylation of the MGLP reducing end *in vivo* (Table 1).

## Discussion

**Figure 7:**
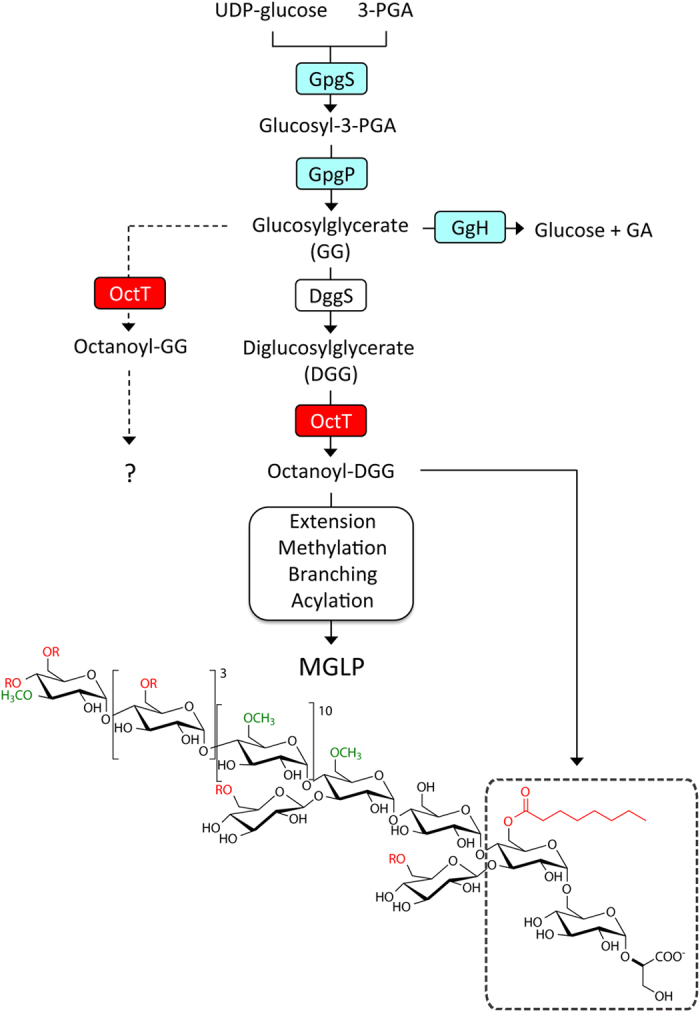
Early steps of the proposed pathway for MGLP biosynthesis. Newly identified octanoyltransferase (OctT) is highlighted red. The dashed line indicates a hypothetical regulatory role of OctT. Experimentally validated functions are shaded blue. Open boxes represent unknown functions or those lacking biochemical confirmation. *R* groups (red) on the structure indicate acyl chains (acetate, propionate, isobutyrate or succinate) and methyl groups are in green. 3-PGA, d-3-phosphoglyceric acid. GpgS, glucosyl-3-phosphoglycerate synthase^21^; GpgP, glucosyl-3-phosphoglycerate phosphatase^23^; DggS, putative diglucosylglycerate synthase^13^ GgH, glucosylglycerate hydrolase (detected in rapidly-growing mycobacteria)^24^.

In this study, we identified a novel mycobacterial acyltransferase that efficiently transfers octanoate to the sugar derivatives glucosylglycerate (GG) and diglucosylglycerate (DGG), the two earliest intermediates in methylglucose lipopolysaccharide (MGLP) biosynthesis^20^ forming its reducing end structure, reported to be octanoylated in the glycerate moiety^14^,^15^. Since our preliminary MS results indicated that OctT did not transfer octanoate to the primary hydroxyl group of glycerate^4^, we deemed essential to perform an exhaustive characterization of the product and found that octanoate is not transferred to glycerate as described for preparations obtained from *M. phlei*^14^, but instead to the C6 OH of the second glucose of DGG (Figs 3 and 7).

The primer for MGLP biosynthesis, glucosyl-3-phosphoglycerate (GPG), is formed from NDP-glucose and 3-phosphoglycerate (3-PGA) by glucosyl-3-phosphoglycerate synthase (GpgS) and dephosphorylated to GG by glucosyl-3-phosphoglycerate phosphatase (GpgP)^21^,^23^. Upon assignment of the second step of MGLP biosynthesis (GG formation) to the GpgP gene *Rv2419c*, its neighboring genes *Rv2417c* and *Rv2418c*, with predicted motifs of lipid-modifying enzymes, were hypothesized to participate in the pathway (Fig. 1)^23^. The moderate level of amino acid identity (24%) between Rv2417c and the *T. maritima* DegV protein (TM841), whose three-dimensional structure was solved with an endogenously co-purified fatty acid chain, could suggest a role in the transport of activated acyl groups or involvement in acyl-CoA synthesis.

A high number of mycobacterial genes are incorrectly annotated, mainly due to the obstacles in performing functional studies arising from the often-difficult purification of the corresponding enzymes from native hosts or from recombinant sources^23^,^47^. Although *Rv2418c* was annotated as a member of the GDSL hydrolase family^31^ based on sequence similarity, this approach has previously proven unreliable for function assignment^48^. A peptidoglycan *O*-acetyltransferase (PatB) with the archetypal catalytic triad Asp-His-Ser of serine esterases annotated as a GDSL hydrolase displayed only weak esterase activity and was shown instead to be a bona-fide acyltransferase^34^. Herein, we show that Rv2418c possesses acyltransferase activity, establishing a previously unrecognized function in mycobacteria.

Based on genetic context and conserved domain analyses, the third step in the MGLP pathway (formation of DGG) was proposed to be catalyzed by Rv3031^13^. However, if the observed substrate ambiguity of OctT toward GG and DGG *in vitro* also occurs *in vivo*, octanoylation of GG in position C6 OH of glucose is expected to curb the progression of MGLP synthesis since this is the site of attachment of the second glucose in DGG and the first unit of the α-(1→4)-linked MGLP main chain^13^. Hypothetically, GG octanoylation *in vivo* could play a regulatory role in the pathway (Fig. 7), possibly related to GG levels accumulated under certain conditions, for example nitrogen starvation. Interestingly, rapidly growing mycobacteria possess a specific GG hydrolase (GgH) activated upon replenishment of assimilable nitrogen to nitrogen-starved cells^24^. However, the mechanism through which mycobacteria tune GG levels while regulating MGLP synthesis and function during exposure to stress remains undisclosed.

Octanoylation is a rare process in nature and several of the octanoyltransferases known to date are involved in fatty acid metabolism. LipB (EC 2.3.1.181) participates in the synthesis of lipoic acid, a highly conserved organosulfur cofactor derived from octanoic acid, which is essential in aerobic bacteria and eukaryotes^49^. The family includes the peroxisomal carnitine *O*-octanoyltransferase (CrOT) (EC 2.3.1.137) responsible for the transfer of medium chain fatty acids to carnitine^50^. Mammalian ghrelin *O*-acyltransferase (GOAT) is a membrane-bound enzyme that transfers octanoate to serine-3 of ghrelin, an appetite-stimulating peptide hormone that only displays activity upon acylation^51^. In yeast, two ethanol *O*-acyltransferases capable of transferring medium-chain fatty acids from CoA to ethanol have been implicated in the biosynthesis of flavor-active ethyl esters, including ethyl octanoate^52^,^53^. Although these are all acyltransferases, the specificity for the acceptor group varies among them, since GOAT and LipB transfer the octanoyl moiety to a specific amino acid residue within a conserved sequence motif or protein domain, respectively, CrOT acylates an ammonium quaternary compound, and ethanol *O*-acyltransferases acylate an alcohol. The OctT identified in this work is, to the best of our knowledge, unique in acylating the sugar derivatives GG and DGG, and in the transfer of short-to-medium chain acyl groups ranging from acetyl to decanoyl, although transfer from the smaller (C2, C3) and larger (C10) acyl chains occurred only at minimal levels.

The *M. smegmatis* OctT had a much higher apparent *k*_cat_ and *k*_cat_/*K*_m_ than the *M. hassiacum* enzyme (Table 2). Very low turnover values (up to 6 × 10^−4^ min^−1^) are known for enzymes referred to as “molecular switches” that signal and regulate shifts between states of activity for transient but defined periods (seconds, hours or longer) favoring accuracy over speed^54^. While it is extremely premature to attribute a signaling role for OctT in the context of MGLP biosynthesis, it is also premature to exclude the contribution of unknown stabilizing factors, post-translational modifications, co-factors or interacting proteins that may translate into higher turnover and catalytic efficiency *in vivo*. One possible explanation for the apparent underperformance of *M. hassiacum* OctT in comparison to the *M. smegmatis* enzyme may reside on the temperature at which the kinetic constants were determined since we decided to study *M. hassiacum* OctT at 37 °C, which is the human host temperature and the optimum for M. smegmatis growth but well below the native host’s optimal growth temperature (50°C)^55^. Despite the similar *k*_cat_/*K*_m_ values for octanoyl and hexanoyl donors (Table 2), the latter has never been detected as a naturally occurring group in MGLP^56^,^57^. Similar promiscuity *in vitro* was reported for GOAT, which is able to use a range of donor acyl chains from acetate to tetradecanoic acid, although octanoylated ghrelin is the predominant form of the enzyme *in vivo* followed by decanoylated ghrelin^58^,^59^. *In vitro,* GOAT prefers hexanoyl-CoA over octanoyl-CoA while decanoyl-CoA is a poor substrate^60^. CrOT also displays fairly broad donor substrate specificity but prefers C6 and C10 substrates^61^. We propose that despite the low-level and apparently broad utilization of acyl-CoAs *in vitro*, OctT may be able to discriminate towards octanoyl groups *in vivo* possibly driven by Oct-CoA availability in the cytoplasm. Narumi and co-workers studied [^14^C]acyl incorporation in *M. phlei* whole cells and found that when [^14^C]hexanoate was supplied to the growth medium it was readily incorporated into MGLP^62^. This allowed them to suggest that the acyl groups found in MGLP likely reflect their intracellular availability in mycobacterial cells at the site of MGLP acylation reactions and also that endogenous hexanoyl-CoA may not be available in the cytoplasm.

Early results by Tung and colleagues with *M. phlei* extracts suggested that distinct MGLP acylating reactions could be performed by a single enzyme, proposed to be capable of transferring acyl groups from different acyl-CoAs to MGLP and similar artificial acceptors^56^. However, the rate of incorporation from succinyl-CoA and octanoyl-CoA was significantly lower than from the acyl-CoAs carrying fatty acids normally esterified to the MGLP non-reducing end (acetate, propionate and isobutyrate), suggesting the involvement of more than one acyltransferase. In support of this hypothesis, we found that succinyl-CoA was not a substrate for OctT, nor did the enzyme efficiently use very short-chain fatty acids (C2-C4) found in the MGLP non-reducing terminus. Although none of the anticipated acyltransferase genes have been so far identified in mycobacterial genomes, genetic context analyses suggest that *Rv3034c* in the cluster containing the 6-*O*-methyltransferase (*Rv3030*) and the 4-*O*-glycosyltransferase (*Rv3032*) genes in *M. tuberculosis* H37Rv might be involved^6^,^13^. Moreover, oligosaccharides mimicking regions of MGLP where acylations have been previously detected could also not be acylated by OctT using different-sized acyl-CoA donors (Table 1). Our results suggest differential acylation of the non-reducing end glucoses and of the branching glucoses in MGLP by enzymes other than OctT, in line with the argument that acylation in such precise locations likely require highly specific acyltransferases^63^.

Two theories regarding MGLP methylation have been put forward: one supports the existence of two different enzymes for differential 6-*O* and 3-*O* methylation of non-reducing end glucoses^6^,^13^. Alternatively, 3-*O*-methylation was suggested to depend on previous acylation^18^. Either because 3-*O*-methylation could depend on previous acylation or on a separate enzyme, an oligosaccharide with (four) unmethylated glucoses could be substrate for an acyltransferase acting on the non-reducing end segment of MGLP. However, and although several unmethylated glucoligosacharides were tested as possible OctT acceptor substrates, acylation from different donors was not detected.

The mycobacterial envelope possesses different surface-exposed acyltrehaloses, namely lipooligosaccharides (LOS) implicated in pathogen-host immune interactions^64^. LOS often differ in the number and structure of sugar residues and the frequently complex acyl moieties attached to trehalose^65^. Evidence suggests that LOS and other antigenic acyltrehaloses may also be esterified with octanoate^3^,^66^,^67^. However, and although we cannot fully exclude an involvement of OctT in these reactions, the insignificant transfer of octanoate to trehalose by OctT renders this a remote possibility.

Herein we report the unprecedented OctT-catalyzed transfer of octanoate to DGG and GG *in vitro* and, to lesser extent, of other short-chain fatty acids. Despite the fact that we cannot fully exclude the existence of an enzyme capable of octanoylating the primary hydroxyl of glyceric acid as anticipated from earlier observations, OctT transfers octanoate to the second glucose in DGG, which may encompass important consequences in MGLP assembly. Since both MGLP and glycogen share a similar α-(1→4)-linked backbone, the formation of octanoate-DGG may discriminate between homofunctional glycosyltransferases and play a critical role in recruiting Rv3032 (the glycosyltransferase proposed to be committed to the elongation) for the MGLP pathway^13^, or the unknown glycosyltransferase for attachment of the first β-(1→3) branching glucose to DGG. However, this remains hypothetical.

Although it is premature to physiologically interpret the results reported here, the proposed essentiality of *Rv2418c* in *M. tuberculosis* renders the knowledge of OctT unique properties valuable in the quest for innovative strategies to fight mycobacterial infections.

## Methods

### Genomic context and sequence analyses

After assignment of the GpgP function to Rv2419c^23^ we sought to investigate the function of the neighboring gene *Rv2418c* (Fig. 1). The Rv2418c amino acid sequence was retrieved from the TubercuList database (http://tuberculist.epfl.ch/index.html)^68^ and used as template for BLAST searches in available mycobacterial genomes. The protein sequence alignment editor Aline^69^ was used for preparing the sequence alignment in Fig. S1.

### Molecular biology and recombinant gene expression

For recombinant expression we selected the gene from *Mycobacterium tuberculosis* H37Rv (*Rv2418c*) and the orthologs from *M. smegmatis* mc^2^155 (MSMEG_4578 or MSMEI_4466 or LJ00_22655) and *M. hassiacum* DSM44199 (GenPept accession WP_026213345.1). Each of the *M. tuberculosis* and *M. hassiacum* sequences were optimized for expression in *E. coli*, synthesized (GenScript) and cloned between the *Nde* I and *Hind* III restriction sites of the expression vector pET30a (Novagen). MSMEI_4466 was amplified from chromosomal DNA purified from *M. smegmatis* (SmartHelix DNAid Mycobacteria kit, Sekvenator, Slovenia). Amplification primers were designed from the sequence retrieved from the SmegmaList database (http://mycobrowser.epfl.ch/smegmalist.html). *Nde* I and *Hind* III restriction sites (underlined) were added to the forward 5′- TACGATCATATGTCCTCTGAGACATCCTCG) and the reverse (5′- TATAAGCTTGGTTGCGGAATCCCGTTG) primers, respectively. Stop codons were removed from reverse primers to allow translation of C-terminal 6 × His-tags. Amplification was carried out in 50 μL mixtures with KOD hot start *Taq* polymerase (Novagen) using 150 ng of template DNA and 68 °C as annealing temperature. PCR products were purified from agarose gels (Jetquick, Genomed) digested with the appropriate restriction enzymes, ligated to the corresponding sites of pET30a (Life Technologies) and transformed into *E. coli* DH5α with standard procedures^70^. Recombinant plasmids were purified and sequenced to confirm the identity of inserts (Macrogen Europe). All constructs were transformed into *E. coli* BL21 and growth was carried out at 37 °C in LB medium containing kanamycin (30 μg/mL) to early exponential phase of growth (OD_610_ = 0.6) in an orbital shaker at 150 rpm. Growth temperature was progressively decreased to 20 °C until mid-exponential phase of growth (OD_610_ = 0.9) and IPTG was added to a final concentration of 0.5 mM to induce gene expression. Cells carrying the recombinant OctT were harvested 18 h later by centrifugation (9000 × *g*, 10 min, 4 °C), suspended in 20 mM sodium phosphate buffer pH 8.0 with 0.3 M NaCl, 10 mM imidazole, 7.5 mM β-mercaptoethanol and 0.1% Tween 20 (buffer A) containing 10 μg/mL DNAse I, and disrupted by sonication on ice with three 40 Hz pulses of 45 s (30 s pause between pulses) per 7 mL of lysate, followed by centrifugation to remove debris (15000 × *g*, 4 °C, 30 min). Protein production was analyzed in 12% SDS-PAGE gels followed by Western immunoblot confirmation. Detection of His-tagged proteins was performed with a 1:1000 dilution (in 3% BSA in TBS) of a mouse anti-His_6_ antibody (Santa Cruz Biotechnology) and a 1:2000 dilution (in 3% (wt/vol) BSA in TBS) of the secondary goat anti-mouse IgG + IgM alkaline phosphatase-conjugated antibody (Bio-Rad). The signal was revealed with Pierce NBT/BCIP 1-Step Solution (Thermo Scientific) and visualized on a Geldoc (Bio-Rad).

### Protein purification

His-tagged recombinant OctTs were purified with a Ni-Sepharose high-performance column (His-Prep FF 16/10, GE Healthcare) equilibrated with buffer A. Elution was carried out in the same buffer containing 200 mM imidazole and the purity of the fractions was evaluated by SDS-PAGE. The purest active fractions pooled after visualization of product formation by thin-layer chromatography (see below) were diluted five-fold with 20 mM Bis-tris propane buffer (BTP) pH 8.0, 200 mM NaCl, 7.5 mM β-mercaptoethanol, 0.1% Tween 20, concentrated by ultrafiltration in 10 kDa molecular weight cutoff centrifugal devices (Amicon) and loaded onto a HiPrep 16/60 Sephacryl S-200 HR column (GE Healthcare) equilibrated with the same buffer and eluted isocratically. The purity of the fractions was assessed by SDS-PAGE and the purest active fractions were pooled, concentrated as described above, and equilibrated in 20 mM BTP pH 8.0, 50 mM NaCl. Protein content was determined by the Bradford assay (Bio-Rad). The molecular mass of the recombinant OctTs was estimated by gel filtration using a HiPrep 16/60 Sephacryl S-200 HR column equilibrated with the 20 mM BTP pH 8.0, 200 mM NaCl, 7.5 mM β-mercaptoethanol, 0.1% Tween 20 using ribonuclease A (13.7 kDa), carbonic anhydrase (29 kDa), ovalbumin (43 kDa) and conalbumin (75 kDa) as molecular mass standards. Blue dextran 2000 (GE Healthcare) was used to determine the void volume.

### Chemical synthesis of GG, DGG and (2*R*)-2-*O*-[6-*O*-octanoyl-(α-d-glucopyranosyl-(1→6)-α-d-glucopyranosyl]-2,3-dihydroxypropanoic acid 1 and of (2*R*)-2-*O*-(α-d-glucopyranosyl-(1→6)-α- d-glucopyranosyl)-3-*O*-octanoyl-2,3-dihydroxypropanoic acid 2

The substrates glucosylglycerate (GG) diglucosylglycerate (DGG) were chemically synthesized as previously described^36^. ^1^H NMR spectra were obtained at 400 MHz in CDCl_3_, DMSO-d_6_ or D_2_O. ^13^C NMR spectra were obtained at 100.61 MHz in the same deuterated solvents. Assignments are supported by 2D correlation NMR studies. Medium pressure preparative column chromatography: Silica Gel Merck 60 H. Analytical TLC: Aluminum-backed Silica Gel Merck 60 F254. Reagents and solvents were purified and dried according to Armarego and Chai^71^. All reactions were carried out under an inert atmosphere (argon) except when the solvents were not dried. The coordinates of all chemically synthesized compounds are indicated as Supplemental Information.

### Ethyl 6-*O*-acetyl-2,3,4-tri-*O*-benzyl-α/β-d-glucopyranosyl-(1→6)-2,3,4-tri-*O*-benzyl-1-thio-α-d-glucopyranoside 5.

A suspension of ethyl 2,3,4-tri-*O*-benzyl-α-1-thio-d-glucopyranoside **3** (0.153 g, 0.309 mmol; Fig. 3A), 6-*O*-acetyl-2,3,4-tri-*O*-benzyl-α/β-d-glucopyranosyl trichloroacetimidate **4** (0.197 g, 0.309 mmol; Fig. 3A)^38^,^39^ and 4Å MS in CH_2_Cl_2_ (3 mL) was stirred at room temperature for 20 minutes. TMSOTf (0.055 mL) was added at −20 °C and the reaction mixture was quenched with saturated NaHCO_3_ aqueous solution (5 mL) after 30 min and extracted with CH_2_Cl_2_ (3 × 10 mL). The combined organic phases were dried with MgSO_4_, filtered and the solvent was removed under vacuum. The crude product was purified by preparative layer chromatography (70/30 hexane/AcOEt). Compound **5** (0.237 g, 79%, α/β = 6:1) was obtained as a colourless viscous foam.

### Ethyl 2,3,4-tri-*O*-benzyl-α-d-glucopyranosyl-(1→6)-2,3,4-tri-*O*-benzyl-1-thio-α-d-glucopyr-anoside 6.

To a solution of compound **5** (0.222 g, 0.229 mmol) in dry methanol, MeONa (5 mg, 0.069 mmol) was added. After 2 hours at room temperature the reaction mixture was quenched with saturated NH_4_Cl aqueous solution (5 mL) and extracted with ethyl acetate (3 × 10 mL). The combined organic phases were dried with MgSO_4_, filtered and the solvent was removed under vacuum. The crude product was purified by preparative layer chromatography (70/30 hexane/AcOEt) to afford compound **6** (0.155 g, 70%, α anomer) as a colourless viscous liquid. The β anomer was also isolated (0.016 g, 8%).

### Ethyl 6-*O*-octanoyl-2,3,4-tri-*O*-benzyl-α/β-d-glucopyranosyl-(1→6)-2,3,4-tri-*O*-benzyl-1-thio-α-d-glucopyranoside 7.

To a solution of compound **6** (0.100 g, 0.108 mmol) in dry CH_2_Cl_2_ (3 mL) DIPEA (34 μL, 0.194 mmol), octanoyl chloride (28 μL, 0.162 mmol) and a catalytic amount of DMAP were added. After 3 hours at room temperature the reaction mixture was quenched with water (5 mL) and extracted with CH_2_Cl_2_ (3 × 10 mL). The combined organic phases were dried with MgSO_4_, filtered and the solvent was removed under vacuum. The crude product was purified by preparative layer chromatography (80/20 hexane/AcOEt). Compound **7** was obtained (0.100 g, 88%) as colourless viscous foam.

### Benzyl 3-*O*-tert-butyldiphenylsilyl-(2R)-2-*O*-[6-*O*-octanoyl-2,3,4-tri-*O*-benzyl-α-d-glucopyra-nosyl-(1→6)-2,3,4-*O*-tri-benzyl-1-thio-α-d-glucopyranosyl]-2,3-dihydroxypropanoate 9.

A suspension of **7** (0.010 g, 0.0095 mmol), benzyl glycerate **8** (0.005 g, 0.309 mmol) and 4Å MS in Et_2_O (3 mL) was stirred at room temperature for 20 minutes. *N*-Iodosuccinimide (0.006 g, 0.012 mmol) and TfOH (7.0 μL of a 0.09M solution) were added at −20 °C. After 30 minutes, 10% Na_2_S_2_O_3_ aqueous solution (5 mL) and saturated NaHCO_3_ aqueous solution (5 mL) were added and the mixture was extracted with CH_2_Cl_2_ (3 × 15 mL). The combined organic phases were dried with MgSO_4_, filtered and the solvent was removed under vacuum. The crude product was purified by preparative layer chromatography (80/20 hexane/AcOEt) to afford **9** (0.011 g, 78%) as a colourless viscous foam.

### Benzyl (2*R*)-2-*O*-[6-O-octanoyl-2,3,4-tri-*O*-benzyl-α-d-glucopyranosyl-(1→6)-2,3,4-tri-*O*- benzyl-α-d-glucopyranosyl]-2,3-dihydroxypropanoate 10.

To a solution of compound **9** (0.035 mg, 0.024 mmol) in dry THF (3 mL) Et_3_N(HF)_3_ (0.737 mmol, 120 μL) was added. The reaction mixture was stirred at room temperature for 48 hours, quenched with water (5 mL) and extracted with ethyl acetate (3 × 10 mL). The combined organic phases were dried with MgSO_4_, filtered and the solvent was removed under vacuum. The crude product was purified by preparative layer chromatography (70/30 hexane/AcOEt) to afford **10** (0.023 g, 81%) as a colourless viscous foam.
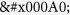


### (2*R*)-2-*O*-[6-*O*-octanoyl-(α-d-glucopyranosyl-(1→6)-α-d-glucopyranosyl]-2,3-dihydroxy-propanoic acid 1.

Compound **10** (0.037 g, 0.031 mmol) in AcOEt (4 mL) was hydrogenated at 50 psi in the presence of Pd/C 10% (0.25 equiv). After 1 hour, the reaction mixture was filtered through Celite and the solvent was removed under vacuum to afford compound **1** (0.017 g, quantitative yield) as a colourless viscous foam (Fig. 2A).

### Benzyl 3-*O*-*tert*-butyldiphenylsilyl-(2*R*)-2-*O*-[2,3-di-*O*-benzyl-4,6-*O*-benzylidene-α-d-glucopyra-nosyl]-2,3-dihydroxypropanoate 12.

A suspension of **11** (Fig. 3B) (0.38 g, 0.7 mmol), benzyl glycerate **8** (0.305 g, 0.7 mmol) and 4 Å MS in CH_2_Cl_2_ was stirred at room temperature for 1 hour. *N*-Iodosuccinimide (0.208 g, 0.92 mmol) and TfOH (4.5 μL) were added at 0 °C. After 50 minutes, 10% (wt/vol) Na_2_S_2_O_3_ aqueous solution (5 mL) and saturated NaHCO_3_ aqueous solution (5 mL) were added and the mixture was extracted with CH_2_Cl_2_ (3 × 15 mL). The combined organic phases were dried (MgSO_4_), filtered, and the solvent was removed under vacuum. The crude product was purified by medium pressure column chromatography (80/20 Hexane/Ethyl acetate) to afford **12** (0.567 g, 93%, α/β = 12:1) as a colourless viscous foam.

### Benzyl 3-*O*-*tert*-butyldiphenylsilyl-(2*R*)-2-*O*-[2,3,4,6-tetra-*O*-benzyl-α-d-glucopyranosyl]-2,3-dihydroxypropanoate 13.

To a solution of **12** (0.21 g, 0.24 mmol) in dry CH_2_Cl_2_ (2.5 mL), 1 M borane solution in THF (1.22 mL, 5 equiv) and TMSOTf (6.6 μL, 0.15 equiv) were added. After 40 min at room temperature 1 mL of Et_3_N was added followed by careful addition of MeOH. The mixture was concentrated and the crude product was dissolved in MeOH (3 × 5 mL) and concentrated again. The crude product was purified by medium pressure column chromatography (90/10 Hexane/Ethyl acetate) to afford **13** (0.09 g, 43%) as a colourless viscous foam.

### Benzyl 3-*O*-*tert*-butyldiphenylsilyl-(2*R*)-2-*O*-[2,3,4,6-tetra-*O*-benzyl-α/β-d-glucopyranosyl-(1→6)-2,3,4-tri-*O*-benzyl-α-d-glucopyranosyl]-2,3-dihydroxypropanoate 15.

A suspension of **13** (0.105 g, 0.12 mmol), compound **14** (0.092 g, 0.13 mmol) and 4 Å MS in CH_2_Cl_2_ was stirred at room temperature for 1 hour. *N*-Iodosuccinimide (0.036 g, 0.16 mmol) and TfOH (3 μL) were added at 0 °C. After 40 minutes, 10% Na_2_S_2_O_3_ aqueous solution (5 mL) and saturated NaHCO_3_ aqueous solution (5 mL) were added and the mixture was extracted with CH_2_Cl_2_ (3 × 15 mL). The combined organic phases were dried (MgSO_4_), filtered and the solvent was removed under vacuum. The crude product was purified by medium pressure column chromatography (80/20 Hexane/Ethyl acetate) to afford **15** (0.14 g, 83%, α/β = 2:1) as a colourless viscous foam.

### Benzyl (2*R*)-2-*O*-[2,3,4,6-tetra-*O*-benzyl-α/β-d-glucopyranosyl-(1→6)-2,3,4-tri-*O*-benzyl-α-d-glucopyranosyl]-2,3-dihydroxypropanoate 16.

To a solution of **15** (0.113 g, 0.08 mmol) in dry THF (5 mL) Et_3_N(HF)_3_ (400 μL) was added and the mixture was stirred at room temperature. After 48 hours the crude product was quenched with water (5 mL) and extracted with ethyl acetate (3 × 10 mL), the combined organic phases were dried (MgSO_4_), filtered and concentrated under vacuum. The crude product was purified by preparative layer chromatography (70/30 Hexane/Ethyl acetate) to afford **16** (0.073 g, 78%, α/β = 3:1) as a colourless viscous foam.

### Benzyl 3-*O*-octanoyl-(2*R*)-2-*O*-[2,3,4,6-tetra-*O*-benzyl-α/β-d-glucopyranosyl-(1→6)-2,3,4-tri-*O*-benzyl-α-d-glucopyranosyl]-2,3-dihydroxypropanoate 17.

To a solution of **16** (0.095 g, 0.08 mmol) in dry CH_2_Cl_2_ (3 mL) DIPEA (27 μL, 0.16 mmol), octanoyl chloride (23 μL, 0.13 mmol) and a catalytic amount of DMAP were added. After 3 hours at room temperature, the mixture was quenched with water (5 mL), extracted with CH_2_Cl_2_ (3 × 10 mL), and the combined organic phases were dried (MgSO_4_), filtered and concentrated under vacuum. The crude product was purified by preparative layer chromatography (70/30 Hexane/Ethyl acetate) to afford **17** (0.092 g, 97%, α/β = 2:1) as a colourless viscous foam.

### (2*R*)-2-*O*-(α-d-glucopyranosyl-(1→6)-α-d-glucopyranosyl)-3-*O*-octanoyl-2,3-dihydroxy-propanoic acid 2.

Compound **17** (0.092 g, 0.07 mmol) in ethyl acetate (10 mL) was hydrogenated at 50 psi in the presence of Pd/C 10% (0.025 equiv). After 6 hours, the reaction mixture was filtered by celite and the solvent was removed under vacuum to afford **2** (0.04 g, 100%) as a colourless viscous foam.

For the detailed experimental procedures for the synthesis of benzyl glycerate **8** and characterization of all the intermediates see Supplemental Information.

### Analysis of enzyme activity by thin-layer chromatography

Enzyme activity was monitored by thin-layer chromatography (TLC) on Silica 60 gel plates (Merck) with a solvent system composed of acetic acid/ethyl acetate/water/ammonia 25% (6:6:2:1, vol/vol). Sugars, sugar derivatives and esterified sugars were visualized by spraying with α-naphtol-sulfuric acid solution followed by charring at 120 °C^72^. Standards of each compound (when available) and of synthetic octanoylated DGG (Oct-DGG) were used for comparative purposes.

### Enzyme assays

The recombinant *M. hassiacum* OctT was selected for biochemical characterization due to its increased stability while the recombinant protein from *M. smegmatis* was used to calculate relevant kinetic constants. Activity was measured with both discontinuous and continuous methods as described below.

For routine detection of activity (continuous assays) in 96-well microtiter plates, the synthetic substrate octanoyl-*p*-nitrophenol (Oct-*p*NP; 1 mM) was added to reaction mixtures containing 50 mM BTP buffer pH 8.0 and 12 mM DGG as acceptor. Reactions (100 μL) were initiated by the addition of enzyme (4 μg) and product formation was followed by monitoring the increase of absorbance at 348 nm, indicative of release of *p*-nitrophenolate. For quantification of reaction rates with CoA-activated substrates (0.5 mM), 2.5 mM of DTNB (5,5′-dithiobis-2-nitrobenzoic acid, Ellman’s Reagent) was added to reaction mixtures prior to enzyme addition, and the release of CoASH was monitored at 412 nm. Control reactions were performed to account for possible substrate degradation and to ensure that ethanol-solubilized DTNB did not influence enzyme activity at the concentrations tested.

*M. hassiacum* OctT temperature and pH profiles were determined with a discontinuous assay in reactions initiated by the addition of 4 μg of OctT to mixtures (100 μL) containing the appropriate buffer, 1 mM Oct-*p*NP and 12 mM DGG. Cooling on an ethanol-ice bath stopped reactions and the enzyme was inactivated by the addition of 2 μL of 5 N HCl. The mixture was neutralized with 2 μL of 5 N NaOH followed by the addition of 100 μL of 100 mM BTP pH 8.0 to stabilize pH. The amount of *p*-nitrophenolate released was estimated by measuring the absorbance of the reaction mixtures at 348 nm. To probe for a possible esterase activity, OctT (4 μg in 100 μL 50 mM BTP pH 8.0) was incubated for 30 min at 37 °C with pure synthetic Oct-DGG (200 μM, 500 μM or 1 mM) with appropriate controls to account for possible degradation.

### Enzyme characterization

Substrate specificity was assessed using glucose, mannose, galactose, sucrose (all from Sigma-Aldrich), laminaribiose (Dextra), trehalose, isomaltose maltose, maltotriose, maltotetraose, maltopentaose, maltohexaose, maltoheptaose (Carbosynth), glucosamine, kanamycin and d,l-glyceric acid (sugars were all tested both independently and in combination with d,l-glyceric acid). GG (α-d-glucosyl-(1→2)-d-glycerate), GPG (α-d-glucosyl-3-phospho-d-glycerate), DGG (α-d-glucosyl-(1→6)-α-d-glucosyl-(1→2)-d-glycerate)^36^, MG (α-d-mannosyl-(1→2)-d-glycerate), MPG (α-d-mannosyl-3-phospho-d-glycerate)^73^, MGG (α-d-mannosyl-(1→2)-α-d-glucosyl-(1→2)-d-glycerate) were also tested as possible acceptors^37^. The CoA derivatives palmitoyl-CoA (C16), tetradecanoyl-CoA (C14), dodecanoyl-CoA (C12), decanoyl-CoA (C10), octanoyl-CoA (C8, Oct-CoA), hexanoyl-CoA (C6, Hex-CoA), succinyl-CoA, butyryl-CoA (C4), propionyl-CoA (C3) and acetyl-CoA (C2) (last three from Larodan Fine Chemicals) were tested as possible acyl donors. Octanoic acid (isolated and in combination with ATP), octanoyl-*p*NP, hexanoyl-*p*NP (TCI), butyryl-*p*NP and acetyl-*p*NP were also tested as possible substrates. All chemicals were from Sigma-Aldrich unless otherwise stated. Pentanoyl-CoA (C5) and heptanoyl-CoA (C7) were not commercially available. The substrate combinations to be tested were selected according to their relevance in the context of MGLP structure and acylation pattern. Product formation was examined by TLC (as described above) and confirmed by MS/MS analysis as described below.

The effect of divalent cations on enzyme activity was examined by incubating the reaction mixture with the chloride salts of Mg^2+^, Mn^2+^, Co^2+^, Zn^2+^ (5 to 20 mM), without cations or in the presence of 10 mM EDTA, at 37 °C. The temperature profile was determined between 25 and 55 °C in 50 mM BTP pH 8.0. The effect of pH was determined at 37 °C in 50 mM buffer (MES (pH 5.5 to 6.5), BTP (pH 6.5 to 9.5) or CAPS (pH 9.5 to 10.0).

### Kinetic parameters

The kinetic parameters for the recombinant OctTs were determined by measuring the amount of CoA released at 412 nm in 96-well microtiter plates as described above. Reactions were performed in 50 mM BTP buffer at 37 °C and initiated by the addition of *M. hassiacum* OctT (4 μg) or *M. smegmatis* OctT (2.5 μg). The *K*_m_ values for DGG, GG, Oct-CoA or Hex-CoA were determined using fixed saturating concentrations of either Oct-CoA or Hex-CoA (100 μM), or DGG and GG (20 mM). The inhibitory effect of free CoA, free octanoate and Oct-DGG product on the reaction rate was tested by the addition of known amounts of these products to mixtures containing fixed concentrations of DGG (20 mM) and Oct-CoA (80 μM).

All experiments were performed in triplicate with appropriate controls. For each method standard curves of *p*-nitrophenol and free CoA were generated to normalize the effect of sugar acceptor and buffer on the absorbance.

### Mass spectrometry

Products of enzymatic reactions (100 μL) containing 50 mM BTP pH 8.0 and 0.5 mM or 1 mM ester donor (CoA or *p*NP), 12 mM sugar acceptor and 4 μg OctT were analyzed by MS or MS/MS. Sample purification was performed with Alltech® Extract-Clean Carbograph columns washed with 50% (vol/vol) and 100% acetonitrile and eluted with 3:1 isopropanol:acetonitrile, 0.1% (vol/vol) trifluoroacetic acid. Eluted reaction products were analyzed by ESI-MS on an Amazon SL ion trap mass spectrometer (Bruker Daltonics, Billerica, MA) at the Mass Spectrometry Facility of the Advanced Analysis Centre (University of Guelph, Canada). Samples were applied by direct infusion at a flow rate of 5 μL/min. The mass spectrometer electrospray capillary voltage was maintained at 4.5 kV and the drying temperature at 300 °C with a flow rate of 8 L/min. Nitrogen was used as both nebulizing (40 psi) and drying gas with helium (60 psi) as collision gas. The mass-to-charge ratio was scanned across the m/z range 15–3000 in enhanced resolution negative-ion mode. The resulting mass spectra were analyzed using the open-source mMass 3.0 software package^74^.

Glyceric acid-containing samples were analyzed by matrix-assisted laser desorption ionization (MALDI) in a time-of-flight (TOF) analyzer at the Proteomics and Mass Spectrometry unit of Institute of Molecular Pathology and Immunology of the University of Porto (IPATIMUP).

### Purification of Oct-DGG and analysis by nuclear magnetic resonance (NMR)

For the analysis of the natural Oct-DGG produced by the *M. tuberculosis*, *M. hassiacum* and *M. smegmatis* recombinant OctT enzymes the product was separated from the reaction components by TLC. Oct-DGG was produced in 20 mL reactions with 50 mM BTP pH 8.0, 7 mM DGG, 1.5 mM Oct-*p*NP or 1.5 mM Oct-CoA and 800 μg of enzyme, for 3 hours at 37 °C. The reactions were spotted on TLC plates and developed with a 2-propanol/ethyl acetate/water/ammonia 25% (5:1:3:1 vol/vol) solvent system. After staining the marginal lanes of the TLC plate and identifying the spot corresponding to Oct-DGG, preparative scale purification of the product was carried out by scraping the corresponding region in the inner unstained lanes of the TLC plate followed by extraction of the product from the silica gel with ultrapure water. The soluble product was then further purified on a Sephadex G10 column (GE Healthcare) with a water flow of 1 mL/min, lyophilized and analyzed by NMR. To confirm the structure of Oct-DGG, with special focus on the position of the octanoate esterification, the ^1^H NMR and ^13^C NMR spectra of synthetic compounds **1** and **2** and of the enzymatically produced one were compared and recorded on a NMR spectrometer Bruker AVANCE II + 400 MHz using D_2_O as solvent.

## Additional Information

**How to cite this article**: Maranha, A. *et al.* Octanoylation of early intermediates of mycobacterial methylglucose lipopolysaccharides. *Sci. Rep.* **5**, 13610; doi: 10.1038/srep13610 (2015).

## Supplementary Material

Supplementary Information

## Figures and Tables

**Table 1:** Substrate specificity of *Mycobacterium hassiacum* OctT towards A) acyl donor B) acceptor substrate C) acyl donors with oligosaccharides mimicking specific acylated motifs found in mature MGLP structure.

A			B		C					
Donor	Acceptor		Acceptor	Donor	Acceptor	Donor				
	GG	DGG		Octanoyl-CoA		Acetyl-CoA	Butyryl-CoA	Succinyl-CoA	Hexanoyl-CoA	Octanoyl-CoA
Acetyl-CoA	−	+	DGG	+++	DGG	+	+	ND	+++	+++
Propionyl-CoA	−	+	GG	++	Trehalose	−	−	−	−	+
Butyryl-CoA	−	++	MGG	+	Maltose	ND	−	ND	−	+
Succinyl-CoA	−	ND	MG	ND	Laminaribiose	−	−	ND	−	ND
Hexanoyl-CoA	++	+++	Glyceric acid	ND	Maltotetraose	ND	−	−	ND	ND
Octanoyl-CoA	++	+++	GPG	ND	Maltopentaose	ND	.ND	−	ND	ND
Decanoyl-CoA	−	+	MPG	ND	Maltoheptaose	−	ND	−	ND	ND
Dodecanoyl-CoA	−	ND	Glucose	ND						
Tetradecanoyl-CoA	−	ND	Mannose	ND						
Palmitoyl-CoA	−	ND	Galactose	ND						
Octanoyl-OH *	−	ND	Trehalose	+						
			Maltose	+						
			Isomaltose	+						
			Sucrose	ND						
			Glucosamine	ND						
			Kanamycin	ND						

ND, Not Detected. +, ++ and +++ indicate increasing amounts of product detected. Product levels + were detected by MS but were not quantifiable with enzymatic assays; Product levels ++ could be spectrophotometrically detected (−) Not tested. When available, *p*NP substrates were also used. *Octanoyl-OH was also tested in combination with ATP with similar results.

**Table 2:** Apparent kinetic parameters of *M. hassiacum* and *M. smegmatis* OctTs.

OctT	Substrate	Co-substrate concentration (fixed)	*K*_m_ (mM)	*V*_*max*_ (nmol/min/mg)	*k*_cat_ (min^−1^)	*k*_*cat*_/*K*_m_ (mM^−1^. min^−1^)
Mh	DGG	Oct-CoA 100 μM	9.5 ± 0.8	134 ± 5	0.1 ± 3 × 10^−3^	0.01 ± 10^−3^
Ms			20.0 ± 3.6	1939 ± 166	2.3 ± 0.2	0.11 ± 0.05
Mh	Oct-CoA	DGG 20 mM	0.06 ± 0.01	162 ± 14	0.12 ± 0.01	1.9 ± 0.4
Ms		DGG 45 mM	0.03 ± 0.01	1898 ± 427	2.2 ± 0.5	86 ± 29
Mh	GG	Oct-CoA 100 μM	6.6 ± 0.78	32 ± 2	0.02 ± 10^−3^	3 × 10^−3^ ± 4 × 10^−4^
Ms			19.0 ± 8.6	20 ± 5	0.02 ± 6 × 10^−4^	0.12 ± 2 × 10^−5^
Mh	Oct-CoA	GG 20 mM	0.01 ± 2 × 10^−3^	21 ± 1	0.01 ± 7 × 10^−4^	1.1 ± 0.2
Ms		GG 45 mM	0.02 ± 2 × 10^−3^	19 ± 3	0.02 ± 3 × 10^−3^	1.5 ± 0.2
Mh	Hex-CoA	DGG 20 mM	0.02 ± 2 × 10^−3^	59 ± 2	0.04 ± 0.01	2.1 ± 0.6
	DGG	Hex-CoA 100 μM	9.8 ± 2.3	41 ± 4	0.03 ± 3 × 10^−3^	3 × 10^−3^ ± 7 × 10^−4^
	Hex-CoA	GG 20 mM	0.01 ± 2 × 10^−3^	19 ± 1	0.01 ± 6 × 10^−4^	1.3 ± 0.2

Mh and Ms represent *M. hassiacum* OctT and *M. smegmatis* OctT, respectively.
