# Supplementary material for: Octanoylation of early intermediates of mycobacterial methylglucose lipopolysaccharides
Source: Sci Rep. 2015 Sep 1;5:13610. doi: 10.1038/srep13610 (PMC4555173; doi:10.1038/srep13610)
Supplement: Supplementary Information [file srep13610-s1.pdf]

Supplemental Information

Octanoylation of early intermediates of mycobacterial methylglucose lipopolysaccharides

Ana Maranha[1], Patrick J. Moynihan[2], Vanessa Miranda[3], Eva Correia Lourenço[3], Daniela Nunes-Costa[1], Joana Fraga[4,5], Pedro José Barbosa Pereira[4,5], Sandra Macedo-Ribeiro[4,5], M. Rita Ventura[3], Anthony J. Clarke[2], Nuno Empadinhas[1,6]*

[1]CNC – Center for Neuroscience and Cell Biology, University of Coimbra, 3004-517 Coimbra, Portugal

[2]Department of Molecular and Cellular Biology, University of Guelph, Ontario, Canada

[3]ITQB – Instituto de Tecnologia Química Biológica, Universidade Nova de Lisboa, Portugal

[4]IBMC – Instituto de Biologia Molecular e Celular, Universidade do Porto, Portugal

[5]Instituto de Investigação e Inovação em Saúde, Universidade do Porto, Portugal

[6]III/UC – Instituto de Investigação Interdisciplinar, University of Coimbra, Portugal

*Correspondence to: Nuno Empadinhas (numenius@cnc.uc.pt)

Supplemental Methods

Chemical synthesis of GG, DGG and (2*R*)-2-*O*-[6-*O*-octanoyl-(α-D-glucopyranosyl-(1→6)-α-D-glucopyranosyl]-2,3-dihydroxypropanoic acid 1 and of (2*R*)-2-*O*-(α-D-glucopyranosyl-(1→6)-α-D-glucopyranosyl)-3-*O*-octanoyl-2,3-dihydroxypropanoic acid 2

Characterisation data for all new compounds:

Ethyl 6-*O*-acetyl-2,3,4-tri-*O*-benzyl-α/β-d-glucopyranosyl-(1→6)-2,3,4-tri-*O*-benzyl-1-thio-α-d-glucopyranoside 5.

FT-IR (ATR): 1740 cm^{-1} (C=O). **^{1}H NMR ($CDCl_3$):** δ 7.35-7.24 (m, Ph), 5.39 (d, J=5.2 Hz, H-1 Glu2 (β,α)), 5.35 (d, J=5.4 Hz, H-1 Glu2 (α,α)), 4.97-4.85 (m, C$\underline{H}_2$Ph), 4.82 (d, J=11.2 Hz, C$\underline{H}_2$Ph (β,α)), 4.78-4.74 (m, C$\underline{H}_2$Ph), 4.71-4.61 (m, C$\underline{H}_2$Ph), 4.58-4.53 (m, C$\underline{H}_2$Ph), 4.49 (d, J=11.3 Hz, C$\underline{H}_2$Ph (β,α)), 4.32-4.30 (m, H-1 Glu1 (β,α)), 4.27-4.24 (m, H-5 Glu2 (α, α)), 4.19 (s, H-6 Glu1(α, α)), 3.96 (t, J=9.2 Hz, H-3 (α, α) Glu1), 3.91-3.84 (m, H-3 Glu2 (α, α), H-5 Glu1 (α, α)), 3.81-3.77 (m, 1xH-6 Glu2 (α, α)), 3.74-3.64 (m, H-2 Glu2 (α, α), 1xH-6 Glu2 (α, α)), 3.57 (t, J=9.5 Hz, H-4 Glu2 (α, α)), 3.51-3.48 (m, H-2 Glu1(α, α)), 3.45 (t, J=9.6 Hz, H-4 (α, α) Glu1), 2.63-2.46 (m, SC$\underline{H}_2CH_3$), 1.99 (s, Ac (β,α)), 1.97 (s, Ac (α, α)), 1.25 (t, J=7.4 Hz, $SCH_2C\underline{H}_3$) ppm. **^{13}C NMR ($CDCl_3$):** δ 170.7 ($\underline{C}OCH_3$), 138.7, 138.6, 138.4, 138.3, 138.1, 137.9, 128.1-127.6, 103.7 (C-1 (β,α) Glu1), 97.0 (C-1 (α,α) Glu1), 82.9 (C-1 (β,α) Glu2), 82.5 (C-3 (α,α) Glu2), 82.4 (C-1 (α,α) Glu2), 81.9 ((β,α)), 81.7 (C-3 (α,α) Glu1), 80.0 (C-2 (α,α) Glu1), 79.8 (C-2 (α,α) Glu2), 79.4 ((β,α)), 77.8 (C-4 (α,α) Glu2), 77.2 (C-4 (α,α) Glu1), 75.7 ($\underline{C}H_2$Ph), 75.6($\underline{C}H_2$Ph), 75.0($\underline{C}H_2$Ph), 74.9($\underline{C}H_2$Ph), 72.6($\underline{C}H_2$Ph), 72.3($\underline{C}H_2$Ph), 70.7 (C-5 (α,α) Glu2), 68.7 (C-5 (α,α) Glu1), 66.4 (C-6 (α,α) Glu2), 63.0 (C-6 (α,α) Glu1), 23.5 ($S\underline{C}H_2CH_3$), 20.9 ($CO\underline{C}H_3$), 14.7 ($SCH_2\underline{C}H_3$) ppm.

Ethyl 2,3,4-tri-*O*-benzyl-α-D-glucopyranosyl-(1→6)-2,3,4-tri-*O*-benzyl-1-thio-α-D-glucopyranoside 6.

α anomer: $[\alpha]_D^{20}$ + 118.0 (c 1.12, CH_2Cl_2). **FT-IR (ATR):** 3451 cm^{-1} (OH) .**^{1}H NMR ($CDCl_3$):** δ 7.35-7.24 (30H, m, Ph), 5.35 (1H, d, J=5.4 Hz, H-1 Glu2), 4.95-4.86 (4H, m, 4xC$\underline{H}$H'Ph), 4.86 (s, H-1 Glu1), 4.78 (1H, d, J=9.6 Hz, CH$\underline{H}$'Ph), 4.75 (1H, d, J=10.4 Hz, CH$\underline{H}$'Ph), 4.67-4.60 (5H, m, 5xC$\underline{H}$H'Ph), 4.56 (1H, d, J=11.7 Hz, CH$\underline{H}$'Ph), 4.25 (1H, dd, J=4.2 Hz, J=10.0 Hz, H-5 Glu2), 3.96 (1H, t, J=9.2 Hz, H-3 Glu1), 3.88 (1H, t, J=9.2 Hz, H-3 Glu2), 3.80 (1H, dd, J=5.1 Hz, J=11.5 Hz, 1xH-6 Glu2), 3.74-3.57 (6H, m, H-5 Glu1, H-6 Glu1, H-2 Glu2, H-4 Glu2, 1xH-6 Glu1), 3.52-3.45 (H-2 Glu1, H-4 Glu1), 2.64-2.47 (2H, m, $SC\underline{H_2}CH_3$), 1.25 (3H, t, J=7.4 Hz, $SCH_2C\underline{H_3}$) ppm. **^{13}C NMR ($CDCl_3$):** δ 138.8, 138.7, 138.4, 138.3, 137.9, 128.4-127.6, 97.2 (C-1 Glu1), 82.6 (C-3 Glu1), 82.5 (C-1 Glu2), 81.6 (C-3 Glu2), 80.2 (C-2 Glu1), 79.8 (C-2 Glu2), 77.7 (C-4 Glu1, C-4 Glu2), 75.7 ($\underline{C}H_2Ph$), 75.6 ($\underline{C}H_2Ph$), 75.0 ($\underline{C}H_2Ph$), 74.9 ($\underline{C}H_2Ph$), 72.6 ($\underline{C}H_2Ph$), 72.3 ($\underline{C}H_2Ph$), 70.9 (C-5 Glu1), 70.8 (C-5 Glu2), 66.4 (C-6 Glu2), 61.9 (C-6 Glu1), 23.6 ($S\underline{C}H_2CH_3$), 14.7 ($SCH_2\underline{C}H_3$) ppm. **HR-MS: calcd**. for $C_{56}H_{62}O_{10}SNa^+$ $[M+Na]^+$: 949.3965; found: 949.3956.

β anomer: [α]D20$[\alpha]_D^{20}$ + 80.6 (c 1.13, CH_2Cl_2). **FT-IR (ATR):** 3486 cm^{-1} (OH). **^{1}H NMR ($CDCl_3$):** δ 7.38-7.15 (30H, m, Ph), 5.39 (1H, d, J=5.2 Hz, H-1 Glu2), 4.95-4.89 (3H, m, C$\underline{H_2}$Ph), 4.84 (1H, d, J=11.0 Hz, C$\underline{H}$H'Ph), 4.81 (1H, d, J=10.9 Hz, C$\underline{H}$H'Ph), 4.76-4.61 (6H, m, C$\underline{H_2}$Ph), 4.49 (1H, d, J=11.3 Hz, C$\underline{H}$H'Ph), 4.35 (1H, d, J=7.8 Hz, H-1 Glu1), 4.24 (1H, d, J=7.8 Hz, H-5 Glu2), 4.07 (1H, d, J=10.7 Hz, 1xH-6 Glu2), 3.88-3.73 (4H, m, H-5 Glu1, 1xH-6 Glu1, H-2 Glu2, 1xH-6 Glu2), 3.67-3.62 (2H, m, H-3 Glu1, 1xH-6 Glu1), 3.54 (2H, t, J=9.2 Hz, H-4 Glu1, H-4 Glu2), 3.48-3.44 (1H, m, H-2 Glu1), 3.35-3.30 (1H, m, H-3 Glu2), 2.60-2.44 (2H, m, $SC\underline{H_2}CH_3$), 1.23 (3H, t, J=7.4 Hz, $SCH_2C\underline{H_3}$) ppm. **^{13}C NMR ($CDCl_3$):** δ 138.8, 138.4, 138.2, 138.0, 137.8, 128.5-127.5, 103.8 (C-1 Glu1), 84.6 (C-3 Glu1), 83.0 (C-1

Glu2), 82.4 (C-5 Glu1), 82.0 (C-2 Glu1), 79.4 (C-2 Glu2), 77.6 (C-4 Glu1, C-4 Glu2), 75.7 ($\underline{C}H_2Ph$), 75.6 ($\underline{C}H_2Ph$), 75.2 (C-3 Glu2), 75.1 ($\underline{C}H_2Ph$), 75.0 ($\underline{C}H_2Ph$), 72.4 ($\underline{C}H_2Ph$), 70.2 (C-5 Glu2), 68.9 (C-6 Glu2), 62.0 (C-6 Glu1), 23.7 ($S\underline{C}H_2CH_3$), 14.7 ($SCH_2\underline{C}H_3$) ppm. **HR-MS: calcd.** for $C_{56}H_{62}O_{10}SNa^+$ $[M+Na]^+$: 949.3968; found: 949.3956.

Ethyl 6-*O*-octanoyl-2,3,4-tri-*O*-benzyl-α/β-D-glucopyranosyl-(1→6)-2,3,4-tri-*O*-benzyl-1-thio-α-D-glucopyranoside 7.

$[\alpha]_D^{20}$ + 110.2 (c 0.91, CH_2Cl_2). **FT-IR (ATR):** 1737 cm^{-1} (C=O). **^1H NMR ($CDCl_3$):** δ 7.35-7.24 (30H, m, Ph), 5.35 (1H, d, J=5.5 Hz, H-1 Glu2), 4.96-4.85 (4H, m, $C\underline{H}H'Ph$), 4.88 (1H, s, H-1 Glu1), 4.77 (1H, d, J=10.7 Hz, $CH\underline{H}'Ph$), 4.76 (1H, m, J=10.8 Hz, $CH\underline{H}'Ph$), 4.68-4.61 (4H, m, $C\underline{H}H'Ph$), 4.56 (1H, d, J=11.7 Hz, $CH\underline{H}'Ph$), 4.53 (1H, d, $CH\underline{H}'Ph$), 4.25 (2H, dd, J=4.7 Hz, J=10.3 Hz, H-5 Glu2), 4.22 (1H, s, H-6 Glu1), 3.96 (1H, t, J=9.2 Hz, H-3 Glu1), 3.91-3.84 (2H, m, H-3 Glu2, H-5 Glu1), 3.80 (1H, dd, J=5.2 Hz, J=11.5 Hz, 1xH-6 Glu2), 3.72 (1H, dd, J=5.5 Hz, J=9.4 Hz, H-2 Glu2), 3.67-3.65 (1H, m, H-6 Glu2), 3.58 (1H, t, J=9.3 Hz, H-4 Glu2), 3.51-3.44 (2H, m, H-2 Glu1, H-4 Glu1), 2.62-2.46 (2H, m, $S\underline{CH_2}CH_3$), 2.25 (2H, t, J=7.5 Hz, $CO\underline{CH_2}(CH_2)_5CH_3$), 1.61-1.55 (2H, m, $COCH_2(C\underline{H_2})_5CH_3$), 1.26-1.23 (11H, m, $COCH_2(C\underline{H_2})_5CH_3$, $SCH_2C\underline{H_3}$), 0.86 (3H, t, J=7.0 Hz, $CO(CH_2)_6C\underline{H_3}$) ppm. **$^{13}C$ NMR ($CDCl_3$):** δ 173.5 ($\underline{C}O(CH_2)_6CH_3$), 138.8, 138.6, 138.4, 138.3, 138.1, 137.9, 128.4-127.6, 97.0 (C-1 Glu1), 82.6 (C-1, Glu2, C-3 Glu1), 81.7 (C-3 Glu1), 80.2 (C-2 Glu1), 79.8 (C-2 Glu2), 77.8 (C-4 Glu2), 77.5 (C-4 Glu1), 75.7 ($\underline{C}H_2Ph$), 75.0 ($\underline{C}H_2Ph$), 72.5 ($\underline{C}H_2Ph$), 72.3 ($\underline{C}H_2Ph$), 70.8 (C-5 Glu2), 68.8 (C-5 Glu1), 66.4 (C-6 Glu2), 62.8 (C-6 Glu1), 34.2 ($CO\underline{C}H_2(CH_2)_5CH_3$), 31.7 ($COCH_2(\underline{C}H_2)_5CH_3$), 29.1 ($COCH_2(\underline{C}H_2)_5CH_3$), 28.9 ($COCH_2(\underline{C}H_2)_5CH_3$), 24.9 ($COCH_2(\underline{C}H_2)_5CH_3$), 23.5 ($S\underline{C}H_2CH_3$), 22.6 ($COCH_2(\underline{C}H_2)_5CH_3$), 14.7 ($SCH_2\underline{C}H_3$), 14.1 ($COCH_2(CH_2)_5\underline{C}H_3$) ppm. **HR-MS:** calcd. for $C_{64}H_{76}O_{11}SNa^+$ $[M+Na]^+$: 1075.0009; found: 1075.0001.

Synthesis of benzyl glycerate 8 and characterization of all intermediates.

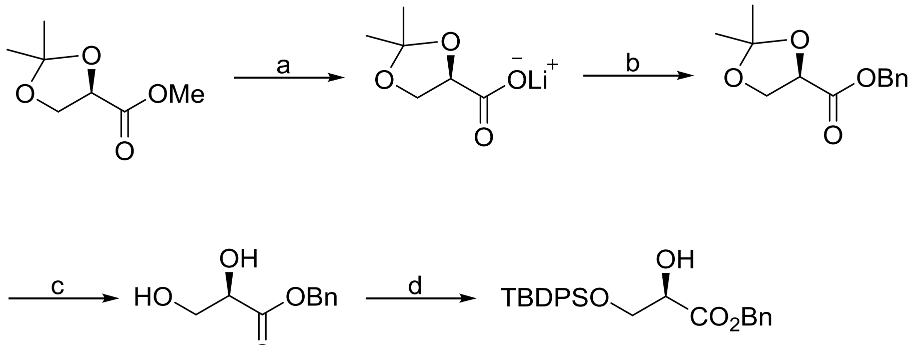

8

a) LiOH (1M), THF:H_2O (4:1), quant. b) BnBr, K_2CO_3, acetone, reflux 70%.
c) Dowex H^+, H_2O:MeOH (2:1), 81%. d) TBDPSCl, DMAP, Pyridine, 60%.

Lithium *O*-isopropylidene-D-glycerate.

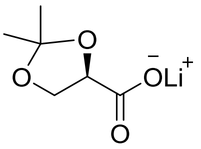

To a solution of initial ester (3.00 g, 18.73 mmol) in THF:H_2O (4:1) a solution of LiOH (1M, 18.73 mmol, 18.7 mL) was added. After 4 hours at room temperature the solvent was removed under vacuum to afford the lithium carboxylate salt (2.850 g, quantitative yield).

$[\alpha]_D^{20}$ + 31.3 (c 1.00, MeOH). **FT-IR (neat):** 1618 (C=O) cm^{-1}. **^{1}H NMR** ($CDCl_3$): δ 4.45 (1H, t, J=7.3 Hz, H-2), 4.22 (1H, t, J=7.8 Hz, H-3), 3.86 (1H, dd, J=6.9 Hz, J=8.3 Hz, H-3), 1.38 (3H, s, CH_3), 1.33 (3H, s, CH_3) ppm. **^{13}C NMR** ($CDCl_3$): δ 177.9 (C=O), 110.7 (O<u>C</u>(CH_3)$_2$O), 75.2 (<u>C</u>H), 66.6 (<u>C</u>H_2), 24.9 (<u>C</u>H_3), 24.5 (<u>C</u>H_3) ppm.

Benzyl *O*-isopropylidene-D-glycerate.

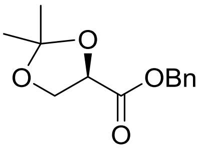

To a solution of the lithium carboxylate salt (3.980 g, 26.17 mmol) in acetone (200 mL) benzyl bromide (52.34 mmol, 6.23 mL) and K_2CO_3 (0.19 mol, 26 g) were added. After 24 hours at reflux the reaction mixture was quenched with water (50 mL) and extracted with ethyl acetate (3x50 mL). The combined organic phases were dried with $MgSO_4$, filtered and the solvent was removed under vacuum to afford the benzyl ester (4.340 g, 70%).

$[\alpha]_D^{20}$ + 16.2 (c 1.05, CH_2Cl_2). **FT-IR (neat):** 1755 (C=O) cm^{-1}. **^{1}H NMR** ($CDCl_3$): δ 7.37-7.35 (5H, m, Ph), 5.23 (1H, d, J=12.2 Hz, $OC\underline{H}_2Ph$), 5.18 (1H, d, J=12.2 Hz, $OC\underline{H}_2Ph$), 4.62 (1H, dd, J=5.1 Hz, J=7.2 Hz, H-2), 4.24 (1H, dd, J=7.2 Hz, J=8.6 Hz, H-3), 4.11 (1H, dd, J=7.2 Hz, J=8.6 Hz, H-3), 1.49 (3H, s, $C\underline{H}_3$), 1.40 (3H, s, $C\underline{H}_3$) ppm. **^{13}C NMR** ($CDCl_3$): δ 171.1 (C=O), 128.6 (Ar), 128.5 (Ar), 128.3 (Ar), 74.1 (C2), 67.3 (C3), 67.0 ($O\underline{C}H_2Ph$), 25.7 ($\underline{C}H_3$), 25.5 ($\underline{C}H_3$) ppm.

Benzyl D-glycerate.

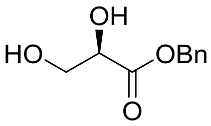

Dowex H^+ (6.321 g) was added to a solution of initial benzyl ester (4.340 g, 18.37 mmol) in H_2O:MeOH (2:1). After 6 hours at room temperature the reaction mixture was filtered and the solvent was removed under vacuum to afford the di-alcohol (2.900 g, 81%).

$[\alpha]_D^{20}$ + 23.7 (c 1.03, CH_2Cl_2). **FT-IR (neat):** 3417 (OH), 1738 (C=O) cm^{-1}. **^{1}H NMR** ($CDCl_3$): δ 7.34-7.30 (5H, m, Ph), 5.22 (1H, d, J=12.2 Hz, $OC\underline{H}_2Ph$), 5.18 (1H, d, J=12.2 Hz, $OC\underline{H}_2Ph$), 4.30 (1H, t, J=3.6 Hz, H-2), 3.89 (1H, dd, J=3.1 Hz, J=11.8 Hz, H-3), 3.83 (1H, dd, J=4.1 Hz, J=11.8 Hz, H-3) ppm. **^{13}C NMR** ($CDCl_3$): δ 172.9 (C=O), 134.9 (C_q - Ar), 128.7 (Ar), 128.6 (Ar), 128.3 (Ar), 71.6 (C2), 67.7 ($O\underline{C}H_2Ph$), 64.0 (C3) ppm.

Benzyl 3-*O*-*tert*-butyldiphenylsilyl-D-glycerate.

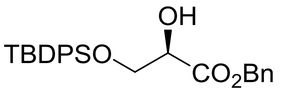

To a solution of initial di-alcohol (2.900 g, 14.78 mmol) in pyridine (15 mL) TBDPSCl (23.46 mmol, 6.45 mL) and a catalytically amount of DMAP were added. After 12 hours at room temperature the reaction mixture was quenched with water (30 mL) and extracted with dichloromethane (3x30 mL). The combined organic phases were dried with ($MgSO_4$), filtered and the solvent was removed under vacuum to afford the benzyl glycerate, compound **8** (3.854 g, 60%).

$[\alpha]_D^{20}$ – 13.43 (c 1.0, CH_2Cl_2). **FT-IR (neat):** 3417 (OH), 1738 cm^{-1} (C=O). **^{1}H NMR** ($CDCl_3$): δ 7.64 (2H, d, J=7.4 Hz, Ph), 7.58 (2H, d, J=7.4 Hz, Ph), 7.42-7.32 (10H, m, Ph), 5.25 (1H, d, J=12.2 Hz, $OC\underline{H}_2Ph$), 5.21 (1H, d, J=12.2 Hz, $OC\underline{H}_2Ph$), 4.27 (1H, s, H-2), 4.01 (1H, dd, J=2.6 Hz, J=10.5 Hz, H-3), 3.92 (1H, dd, J=2.8 Hz, J=10.5 Hz, H-3), 1.01 (9H, s, TBDPS) ppm. **^{13}C NMR** ($CDCl_3$): δ 172.8 (C=O), 135.6 (Ar), 135.5 (Ar), 132.9 (C_q - Ar), 132.8 (C_q - Ar), 129.9 (Ar), 129.8 (Ar), 128.7 (Ar), 128.5 (Ar), 128.4 (Ar), 127.8 (Ar), 127.7 (Ar), 72.0 (C2), 67.4 ($O\underline{C}H_2Ph$), 65.8 (C3), 26.7 ($\underline{C}H_3$ - *t*Bu), 19.3 (C_q - *t*Bu) ppm.

Benzyl 3-*O*-*tert*-butyldiphenylsilyl-(2R)-2-*O*-[6-*O*-octanoyl-2,3,4-tri-*O*-benzyl-α-D-glucopyranosyl-(1→6)-2,3,4-*O*-tri-benzyl-1-thio-α-D-glucopyranosyl]-2,3-dihydroxypropanoate 9.

$[\alpha]_D^{20}$ + 67.6 (c 1.02, CH_2Cl_2). FT-IR (ATR): 1737 cm-1 (C=O). **^{1}H NMR ($CDCl_3$):** δ 7.71-7.64 (4H, m, Ph), 7.35-7.17 (40H, m, Ph), 5.20 (1H, d, J=12.2 Hz, $CO_2C\underline{H}HPh$), 5.19 (1H, d, J=3.5 Hz, H-1 Glu2), 5.12 (1H, d, J=12.3 Hz, $CO_2CH\underline{H'}Ph$), 4.98-4.90 (3H, m, $C\underline{H}H'Ph$), 4.93 (1H, s, H-1 Glu1), 4.85 (1H, d, J=10.8 Hz, $C\underline{H}H'Ph$), 4.79 (1H, d, J=11.6 Hz, $C\underline{H}H'Ph$), 4.75 (1H, d, J=10.7 Hz, $CH\underline{H}'Ph$), 4.74 (1H, d, J=10.8 Hz, $CH\underline{H}'Ph$), 4.63 (1H, d, J=10.8 Hz, $CH\underline{H}'Ph$), 4.57-4.48 (5H, m, 4x $C\underline{H}H'Ph$, $CH_2C\underline{H}(OTBDPS)CO_2Bn$), 4.16 (1H, s, 1xH-6

Glu1), 4.09-4.05 (2H, m, H-3 Glu2, 1x$C\underline{H}_2CH(OTBDPS)CO_2Bn$), 3.99 (1H, dd, J=3.4 Hz, J=10.7 Hz, 1x$C\underline{H}_2CH(OTBDPS)CO_2Bn$), 3.93 (1H, t, J=9.2 Hz, H-3 Glu1), 3.86-3.84 (1H, m, H-5 Glu2), 3.79-3.72 (3H, m, H-4 Glu2, H-5 Glu1, 1xH-6 Glu2), 3.56-3.42 (4H, m, H-2 glu2, 1xH-6 Glu2, H-2 Glu1, H-4 Glu1), 2.22 (2H, t, J=7.9 Hz, $COC\underline{H_2}(CH_2)_5CH_3$), 1.24-1.23 (10H, m, $COCH_2(C\underline{H_2})_5CH_3$), 1.00 (9H, s, $SiC(\underline{C}H_3)_3$), 0.85 (3H, t, J=7.0 Hz, $COCH_2(CH_2)_5C\underline{H_3}$) ppm. **^{13}C NMR ($CDCl_3$):** δ 173.5 ($\underline{C}O(CH_2)_6CH_3$), 169.7 ($\underline{C}O_2CH_2Ph$), 138.9, 138.7, 138.6, 138.3, 138.2, 138.1, 135.7 (Ph), 135.5 (Ph), 135.4 (Ph), 133.1, 132.8, 129.7 (Ph), 128.6-127.5 (Ph), 97.2 (C-1 Glu1), 94.8 (C-1 Glu2), 81.7 (C-3 Glu2), 81.6 (C-3 Glu1), 79.9 (C-2 Glu1), 79.5 (C-2 Glu2), 77.1 (C-4 Glu2, C-4 Glu1), 75.7 ($\underline{C}H_2Ph$), 75.6 ($\underline{C}H_2Ph$), 75.0 ($\underline{C}H_2Ph$), 74.8 ($\underline{C}H_2Ph$), 74.6 ($CH_2\underline{C}H(OTBDPS)CO_2Bn$), 72.1 ($\underline{C}H_2Ph$), 71.8 ($\underline{C}H_2Ph$), 71.0 (C-5 Glu2), 68.8 (C-5 Glu1), 66.8 ($CO_2\underline{C}H_2Ph$), 65.6 (C-6 Glu2), 64.9 ($\underline{C}H_2CH(OTBDPS)CO_2Bn$), 62.7 (C-6 Glu1), 34.1 ($CO\underline{C}H_2(CH_2)_5CH_3$), 31.6 ($COCH_2(\underline{C}H_2)_5CH_3$), 29.1 ($COCH_2(\underline{C}H_2)_5CH_3$), 28.9 ($COCH_2(\underline{C}H_2)_5CH_3$), 26.7 ($SiC(\underline{C}H_3)_3$), 24.8 ($COCH_2(\underline{C}H_2)_5CH_3$), 22.6 ($COCH_2(\underline{C}H_2)_5CH_3$), 19.2 ($Si\underline{C}(CH_3)_3$), 14.1 ($COCH_2(CH_2)_5\underline{C}H_3$) ppm. **HR-MS**: calcd. for $C_{88}H_{100}O_{15}SiNa^+$ $[M+Na]^+$: 1447.6724; found: 1447.6724.

Benzyl (2*R*)-2-*O*-[6-O-octanoyl-2,3,4-tri-*O*-benzyl-α-D-glucopyranosyl-(1→6)-2,3,4-tri-*O*-benzyl-α-D-glucopyranosyl]-2,3-dihydroxypropanoate 10.

$[\alpha]_D^{20}$ + 75.7 (c 0.885, CH_2Cl_2). **FT-IR (ATR)**: 3417 (OH), 1738 (C=O) cm^{-1}. **^{1}H NMR ($CDCl_3$):** δ 7.71-7.64 (m, Ph), 7.35-7.17 (m, Ph), 5.20 (1H, d, J=12.2 Hz, $CO_2C\underline{H}H'Ph$), 5.19 (1H, d, J=3.5 Hz, H-1 Glu2), 5.12 (1H, d, J=12.3 Hz, $CO_2CH\underline{H}'Ph$), 4.99 (1H, d, J=10.8 Hz, $C\underline{H}H'Ph$), 4.91 (1H, d, J=10.8 Hz, $C\underline{H}H'Ph$), 4.90 (1H, d, J=11.0 Hz, $C\underline{H}H'Ph$), 4.85 (1H, d, J=10.8 Hz, $C\underline{H}H'Ph$), 4.80-4.74 (3H, m, $CH\underline{H}'Ph$), 4.93 (1H, s, H-1 Glu1), 4.70 (1H, d, J=12.2 Hz, $C\underline{H}H'Ph$), 4.60 (1H, d, J=13.7 Hz, $CH\underline{H}'Ph$), 4.55 (1H, d, J=11.8 Hz, $CH\underline{H}'Ph$),

4.56 (1H, d, J=8.4 Hz, $C\underline{H}H'Ph$), 4.52 (1H, d, J=10.8 Hz, $CH\underline{H}'Ph$), 4.45 (1H, dd, J=3.4 Hz, J=6.1 Hz, $CH_2C\underline{H}(OH)CO_2Bn$), 4.21 (2H, d, J=3.0 Hz, H-6 Glu1), 4.11-4.07 (1H, m, H-5 Glu2), 4.04 (1H, t, J=9.2 Hz, H-3 Glu2), 3.99-3.92 (3H, m, $C\underline{H_2}CH(OH)CO_2Bn$, H-3 Glu1), 3.82-3.76 (1H, m, H-5 Glu1), 3.70 (1H, dd, J=6.3 Hz, J=11.2 Hz, 1xH-6 Glu1), 3.63-3.51 (3H, m, 1xH-6 Glu1, H-4 Glu2, H-2 Glu2), 3.50-3.44 (2H, m, H-2 Glu1, H-4 Glu1), 2.27-2.23 (2H, m, $COC\underline{H_2}(CH_2)_5CH_3$), 1.57 (2H, t, J=7.2 Hz, $COCH_2(C\underline{H_2})_5CH_3$), 1.25-1.24 (8H, m, $COCH_2(C\underline{H_2})_5CH_3$), 0.85 (3H, t, J=7.0 Hz, $COCH_2(CH_2)_5C\underline{H_3}$) ppm. **^{13}C NMR ($CDCl_3$)**: δ 173.5 ($\underline{C}O(CH_2)_6CH_3$), 169.6 ($\underline{C}O_2CH_2Ph$), 138.8, 138.5, 138.1, 138.0, 137.9, 137.8, 135.3, 128.6-127.6 (Ph), 97.4 (C-1 Glu1), 95.0 (C-1 Glu2), 81.9 (C-3 Glu1), 81.8 (C-3 Glu2), 79.9 (C-4 Glu1), 79.5 (C-4 Glu2), 77.7 (C-2 Glu2), 77.4 (C-2 Glu2), 75.8 ($\underline{C}H_2Ph$), 75.7 ($\underline{C}H_2Ph$), 75.2 ($CH_2\underline{C}H(OH)CO_2Bn$), 75.1 ($\underline{C}H_2Ph$), 73.0 ($\underline{C}H_2Ph$), 72.2 ($\underline{C}H_2Ph$), 70.7 (C-5 Glu2), 68.9 (C-5 Glu1), 66.9 (C-6 Glu2), 66.5 ($CO_2\underline{C}H_2Ph$), 63.4 ($\underline{C}H_2CH(OH)CO_2Bn$), 62.6 (C-6 Glu1), 34.1 ($CO\underline{C}H_2(CH_2)_5CH_3$), 31.6 ($COCH_2(\underline{C}H_2)_5CH_3$), 29.1 ($COCH_2(\underline{C}H_2)_5CH_3$), 28.9 ($COCH_2(\underline{C}H_2)_5CH_3$), 24.9 ($COCH_2(\underline{C}H_2)_5CH_3$), 22.6 ($COCH_2(\underline{C}H_2)_5CH_3$), 14.1 ($COCH_2(CH_2)_5\underline{C}H_3$) ppm. **HR-MS**: calcd. for $C_{72}H_{82}O_{15}Na^+$ $[M+Na]^+$: 1209.5546; found: 1209.5577.

(2*R*)-2-*O*-[6-*O*-octanoyl-(α-D-glucopyranosyl-(1→6)-α-D-glucopyranosyl]-2,3-dihydroxypropanoic acid 1.

$[\alpha]_D^{20}$ + 88.9 (c 0.73, H_2O). **FT-IR (ATR):** 3356 (OH), 1633 (C=O) cm^{-1}. **^{1}H NMR (D_2O):** δ 4.95 (1H, d, J=3.6 Hz, H-1 Glu1), 4.86 (1H, d, J=3.5 Hz, H-1 Glu2), 4.38 (1H, d, J=10.6 Hz, 1xH-6 Glu1), 4.19 (1H, dd, J=5.9 Hz, J=12.1 Hz, 1xH-6 Glu1), 4.12 (1H, dd, J=3.0 Hz, J=5.9 Hz, $CH_2C\underline{H}(OH)CO_2H$), 3.90-3.83 (2H, m, 1xH-6 Glu2, H-5 Glu1), 3.81-3.72 (3H, m, $C\underline{H_2}CH(OH)CO_2H$, H-5 Glu2), 3.69-3.65 (3H, m, H-3 Glu2, 1xH-6 Glu2, H-3 Glu1), 3.51-3.46 (2H, m, H-2 Glu1, H-2 Glu2), 3.42 (1H, t, J=9.5 Hz, H-4 Glu2), 3.35 (1H, J=9.8 Hz, H-4

Glu1), 2.35 (2H, t, J=7.3 Hz, $COC\underline{H_2}(CH_2)_5CH_3$), 1.55 (2H, t, J=7.2 Hz, $COCH_2(C\underline{H_2})_5CH_3$), 1.26-1.20 (8H, m, $COCH_2(C\underline{H_2})_5CH_3$), 0.81-0.77 (3H, m, $COCH_2(CH_2)_5C\underline{H_3}$) ppm. **^{13}C NMR ($CDCl_3$)**: δ 176.9 (C=O), 176.8 (C=O), 97.7 (C-1 Glu2), 97.5 (C-1 Glu1), 79.3 ($CH_2\underline{C}H(OH)CO_2H$), 73.5 (C-3 Glu2), 72.9 (C-3 Glu1), 71.6 (C-2 Glu2), 71.4 (C-2 Glu1), 70.6 (C-5 Glu1), 69.8 (C-4 Glu1), 69.7 (C-4 Glu2), 69.5 (C-5 Glu2), 65.7 (C-6 Glu2), 63.2 (C-6 Glu1), 63.1 ($\underline{C}H_2CH(OH)CO_2H$), 33.8 ($CO\underline{C}H_2(CH_2)_5CH_3$), 30.9 ($COCH_2(\underline{C}H_2)_5CH_3$), 28.2 ($COCH_2(\underline{C}H_2)_5CH_3$), 28.0 ($COCH_2(\underline{C}H_2)_5CH_3$), 24.3 ($COCH_2(\underline{C}H_2)_5CH_3$), 22.0 ($COCH_2(\underline{C}H_2)_5CH_3$), 13.4 ($COCH_2(CH_2)_5\underline{C}H_3$) ppm. **HR-MS**: calcd. for $C_{23}H_{40}O_{15}Na^+$ $[M+Na]^+$: 579.2249; found: 579.2259.

Benzyl 3-*O*-*tert*-butyldiphenylsilyl-(2*R*)-2-*O*-[2,3-di-*O*-benzyl-4,6-*O*-benzylidene-α-D-glucopyranosyl]-2,3-dihydroxypropanoate 12.

$[\alpha]^{20}_{D}[\alpha]_{D}^{20}$ + 31.9 (c 1.1, CH_2Cl_2)**. FT-IR (neat):** 1749 (C=O) cm^{-1}. **^{1}H NMR ($CDCl_3$):** δ 7.70-7.57 (m, Ph), 7.44-7.20 (m, Ph), 5.51 (s, $PhC\underline{H}$ (α)), 5.50 (s, $PhC\underline{H}$ (β)), 5.23-5.18 (m, $CO_2C\underline{H_2}Ph$, H-1 (α)), 4.88-4.81 (m, $C\underline{H_2}Ph$ (α)), 4.88-4.84 (m, $C\underline{H_2}Ph$ (α)), 4.66 (d, J=11.8 Hz, $C\underline{H_2}Ph$ (α)), 4.58 (d, J=7.2 Hz, H-1 (β)), 4.50 (dd, J=3.9 Hz, J=5.8 Hz, $CH_2C\underline{H}(OTBDPS)CO_2Bn$ (α)), 4.16 (dd, J=4.8 Hz, J=10.2 Hz, 1xH-6), 4.11-4.04 (m, $1xC\underline{H_2}CH(OTBDPS)CO_2Bn$, H-3 (α)), 4.03-3.98 (m, $1xC\underline{H_2}CH(OTBDPS)CO_2Bn$ (α)), 3.94-3.88 (m, H5 (α)), 3.67-3.57 (m, H-2, H-4, H-6 (α)), 1.01 (s, $SiC(C\underline{H_3})_3$) ppm. **^{13}C NMR ($CDCl_3$):** δ 169.6 (C=O), 138.8, 138.2, 137.4, 135.7-135.5 (Ph), 135.3, 133.0, 132.8, 129.7-126.2 (Ph), 101.4 ($Ph\underline{C}H$), 95.9 (C-1 (α)), 81.8 (C-4 (α)), 78.7 (C-2 (α)), 77.9 (C-3 (α)), 75.2 ($\underline{C}H_2Ph$), 74.9 ($CH_2\underline{C}H(OTBDPS)CO_2Bn$ (α)), 72.1 ($\underline{C}H_2Ph$), 68.8 (C-6 (α)), 66.9 ($\underline{C}H_2Ph$), 64.7 ($\underline{C}H_2CH(OTBDPS)CO_2Bn$ (α)), 62.9 (C-5 (α)), 26.7 ($SiC(\underline{C}H_3)_3$), 19.2 ($Si\underline{C}(CH_3)_3$) ppm.

Benzyl 3-*O*-*tert*-butyldiphenylsilyl-(2*R*)-2-*O*-[2,3,4,6-tetra-*O*-benzyl-α-D-glucopyranosyl]-2,3-dihydroxypropanoate 13.

$[\alpha]_D^{20}[\alpha]_D^{20}$ + 63.9 (c 0.9, CH_2Cl_2). **FT-IR (neat):** 1745 (C=O) cm^{-1}. **^{1}H NMR ($CDCl_3$):** δ 7.69-7.65 (4H, m, Ph), 7.38-7.23 (26H, m, Ph), 5.23 (1H, d, J=12.2 Hz, $CO_2C\underline{H}H'Ph$), 5.19 (1H, d, J=3.4 Hz, H-1), 5.16 (1H, d, J=12.3 Hz, $CO_2CH\underline{H}'Ph$), 4.99 (1H, d, J=10.8 Hz, $C\underline{H}H'Ph$ (A)), 4.88 (1H, d, J=11.4 Hz, $C\underline{H}H'Ph$ (B)), 4.84 (1H, d, J=11.8, $C\underline{H}H'Ph$ (C)), 4.77 (1H, d, J=10.8 Hz, $CH\underline{H}'Ph$ (A)), 4.62 (1H, d, J=11.2 Hz, $CH\underline{H}'Ph$ (B)), 4.60 (1H, d, J=11.2 Hz, $CH\underline{H}'Ph$ (C)), 4.50-4.48 (1H, m, $CH_2C\underline{H}(OTBDPS)CO_2Bn$), 4.11-3.98 (3H, m, $C\underline{H_2}CH(OTBDPS)CO_2Bn$, H-3), 3.79-3.77 (1H, m, H-5), 3.67-3.51 (4H, m, H-2, H-4, H-6), 1.01 (9H, s, $SiC(C\underline{H_3})_3$) ppm. **^{13}C NMR ($CDCl_3$):** δ 169.7 ($\underline{C}$=O), 138.9, 138.4, 138.2, 135.7, 135.6, 135.4, 133.1, 132.8, 129.8-127.6 (Ph), 94.9 (C-1), 81.5 (C-3), 79.3 (C-2), 77.1 (C-4), 75.7 ($\underline{C}H_2Ph$), 74.9 ($\underline{C}H_2Ph$), 74.8 ($CH_2\underline{C}H(OTBDPS)CO_2Bn$), 71.9 ($\underline{C}H_2Ph$), 71.3 (C-5), 66.9 ($CO_2C\underline{H_2}Ph$), 64.8 ($\underline{C}H_2CH(OTBDPS)CO_2Bn$), 61.7 (C-6), 26.8 ($SiC(\underline{C}H_3)_3$), 19.2 ($Si\underline{C}(CH_3)_3$) ppm.

Benzyl 3-*O*-*tert*-butyldiphenylsilyl-(2*R*)-2-*O*-[2,3,4,6-tetra-*O*-benzyl-α/β-D-glucopyranosyl-(1→6)-2,3,4-tri-*O*-benzyl-α-D-glucopyranosyl]-2,3-dihydroxypropanoate 15.

FT-IR (neat): 1738 (C=O) cm^{-1}. **^{1}H NMR ($CDCl_3$):** δ 7.70-7.64 (m, Ph), 7.37-7.08 (m, Ph), 5.28 (d, J=3.4 Hz, H-1 Glu2 (β, α)), 5.27-5.10 (m, $C\underline{H_2}Ph$), 5.21 (s, H-1 Glu2 (α, α)), 4.99-4.87 (m, $C\underline{H_2}Ph$), 4.94 (s, H-1 Glu1 (α, α)), 4.84-4.60 (m, $C\underline{H_2}Ph$), 4.57-4.52 (m, $C\underline{H_2}Ph$, $CH_2C\underline{H}(OTBDPS)CO_2Bn$), 4.49-4.37 (m, $C\underline{H_2}Ph$), 4.25 (d, J=7.8 Hz, H-1 Glu1 (β, α)), 4.12-3.98 (m, H-5 Glu2 (α, α), $C\underline{H_2}CH(OTBDPS)CO_2Bn$, H-6 Glu1 (α, α)), 3.93-3.74 (m, H-3 Glu1 (α, α), H-3 Glu2 (α, α), H-6 Glu2 (α, α)), 3.68-3.47 (m, H-5 Glu1 (α, α), H-2 Glu1 (α, α), H-4 Glu1 (α, α), H-2 Glu2 (α, α), H-4 Glu2 (α, α), H'-6 Glu2 (α, α)), 3.45-3.37 (H-2 (β, α),

H-4 (β, α)), 1.00 (bs, $SiC(C\underline{H_3})_3$) ppm. **^{13}C NMR ($CDCl_3$):** δ 169.8 ($\underline{C}O_2Bn$ (α, α)), 169.7 ($\underline{C}O_2Bn$ (β, α)), 139.0-138.0, 135.7 (α, α), 135.6 (β,α), 135.5 (α, α), 135.4 (β,α), 133.2 (α, α), 133.1 (β,α), 132.8 (α, α), 129.7, 128.6-127.3, 103.7 (C-1 Glu1 (β,α)), 97.5 (C-1 Glu1 (α,α)), 94.8 (C-1 Glu2 (α,α)), 94.7 C-1 Glu2 (β,α)), 84.8 (β,α), 82.1 (β,α), 81.7 (α,α), 81.5 (β,α), 79.9 (α,α), 79.5 (α,α), 79.0 (β,α), 77.9 (β,α), 77.6 (α,α), 77.1 (α,α), 75.7 (β,α), 75.6 (α,α), 75.5 (α,α), 75.1 ($CH_2\underline{C}H(OTBDPS)CO_2Bn$ (β, α)), 75.0 (α,α), 74.9 (α,α), 74.8 (α,α), 74.7 (β,α), 74.6 ($CH_2\underline{C}H(OTBDPS)CO_2Bn$ (α, α)), 73.4 (α, α), 72.2 (α, α), 71.8 (α, α), 71.7 (β,α), 71.1 (α,α), 70.3 (α,α), 70.2 (β,α), 69.0 (β,α), 68.4 (α,α), 66.8 (α,α), 66.7 (β,α), 65.7 (α,α), 64.9 (α,α), 26.8 ($SiC(\underline{C}H_3)_3$ (β, α)), 26.7 ($SiC(\underline{C}H_3)_3$ (α, α)), 19.2 ($Si\underline{C}(CH_3)_3$ (α, α)) ppm.

Benzyl (2*R*)-2-*O*-[2,3,4,6-tetra-*O*-benzyl-α/β-D-glucopyranosyl-(1→6)-2,3,4-tri-*O*-benzyl-α-D-glucopyranosyl]-2,3-dihydroxypropanoate 16.

FT-IR (neat): 3420 (OH), 1739 (C=O) cm^{-1}. **^{1}H NMR ($CDCl_3$):** δ 7.31-7.10 (m, Ph), 5.29-5.26 (m, $C\underline{H_2}Ph$), 5.26 (bs, H-1 Glu2 (β,α)), 5.22 (d, J=12.2 Hz, C$\underline{H}$H'Ph), 5.13-5.10 (m, $C\underline{H_2}Ph$), 5.12 (H-1 Glu2 (α, α)), 5.00-4.96 (m, $C\underline{H_2}Ph$), 4.92-4.87 (m, $C\underline{H_2}Ph$), 4.84-4.69 (m, $C\underline{H_2}Ph$), 4.76 (bs, H-1 Glu1 (α, α)), 4.61-4.41 (m, $C\underline{H_2}Ph$, $CH_2C\underline{H}(OTBDPS)CO_2Bn$), 4.32 (d, J=7.8 Hz, H-1 Glu1 (β,α)), 4.16-4.11 (m), 4.06-3.87 (m), 3.75-3.49 (m), 3.47-3.46 (H-2(β,α)) ppm. **^{13}C NMR ($CDCl_3$):** δ 169.7 ($\underline{C}O_2Bn$ (α, α)), 169.5 ($\underline{C}O_2Bn$ (β, α)), 138.8 (β,α), 138.7 (α, α), 138.5 (β,α), 138.3 (α, α), 138.2 (α, α), 138.1 (β,α), 138.0 (α, α), 137.9 (α, α), 137.8 (β,α), 135.3 (α, α), 135.2 (β,α), 128.6-127.5 (Ph), 103.8 (C-1 Glu1 (β,α)), 97.6 (C-1 Glu1 (α,α)), 95.0 (C-1 Glu2 (α,α)), 94.9 (C-1 Glu2 (β,α)), 84.8 (β,α), 82.1 (α, α), 82.0 (β,α), 81.8 (α, α), 81.3 (β,α), 79.8 (α, α), 79.5 (α, α), 79.0 (β,α), 77.8 (α, α), 77.6 (α, α), 75.7 (α, α), 75.6 (β,α), 75.3 (β,α), 75.1 ($CH_2\underline{C}H(OTBDPS)CO_2Bn$ (α, α)), 75.0 (α, α), 74.9 (β,α), 74.8 (β,α), 74.6 ($CH_2\underline{C}H(OTBDPS)CO_2Bn$ (β, α)), 73.4 (α, α), 73.0 (α, α), 72.1 (α, α), 70.7 (β, α), 70.6 (α, α), 70.3 (α, α), 68.9 (C-6 (β, α)), 68.5 (C-6 (β, α)), 68.3 (C-6 (α, α)), 66.9 ($CO_2\underline{C}H_2Ph$

(α, α)), 66.5 (C-6 (α, α)), 63.4 ($\underline{C}H_2CH(OTBDPS)CO_2Bn$ (β, α)), 63.3 ($\underline{C}H_2CH(OTBDPS)CO_2Bn$ (α, α)) ppm.

Benzyl 3-*O*-octanoyl-(2*R*)-2-*O*-[2,3,4,6-tetra-*O*-benzyl-α/β-D-glucopyranosyl-(1→6)-2,3,4-tri-*O*-benzyl-α-D-glucopyranosyl]-2,3-dihydroxypropanoate 17.

FT-IR (neat): 1748 and 1739 (C=O) cm^{-1}. **^{1}H NMR ($CDCl_3$):** δ 7.31-7.10 (m, Ph), 5.27-5.22 (m, $C\underline{H_2}Ph$), 5.24 (bs, H-1 Glu2 (β,α)), 5.18-5.10 (m, $C\underline{H_2}Ph$), 5.16 (d, J=3.5 Hz, H-1 Glu2 (α, α)), 5.03-4.88 (m, CH_2Ph), 4.98 (bs, H-1 Glu1 (α, α)), 4.82-4.70 (m, $C\underline{H_2}Ph$), 4.67-4.38 (m, $C\underline{H_2}Ph$, $CH_2C\underline{H}(OH)CO_2Bn$), 4.29 (d, J=7.7 Hz, H-1 Glu1 (β,α)), 4.19 (d, J=9.2 Hz (β,α)), 4.03-3.83 (m), 3.74-3.47 (m), 3.45 (bs (β,α)), 3.42-3.39 (m (β,α)), 2.23-2.20 (m, $COC\underline{H_2}(CH_2)_5CH_3$), 1.54-1.50 (m, $COCH_2(C\underline{H_2})_5CH_3$), 1.20 (bs, $COCH_2(C\underline{H_2})_5CH_3$), 0.84 (t, J=7.0 Hz, $COCH_2(CH_2)_5C\underline{H_3}$) ppm. **^{13}C NMR ($CDCl_3$):** δ 173.2 ($\underline{C}OCH_2(CH_2)_5CH_3$), 168.9 ($\underline{C}O_2Bn$ (α, α)), 168.8 ($\underline{C}O_2Bn$ (β, α)), 138.9 (β, α), 138.8 (α, α), 138.7 (α, α), 138.5 (α, α), 138.4 (α, α), 138.4 (β, α), 138.3 (β, α), 138.2 (β, α), 138.1 (β, α), 138.0 (α, α), 137.9 (α, α), 135.1 (α, α), 135.0 (β, α), 128.6-127.4 (Ph), 103.7 (C-1 Glu1 (β,α)), 97.4 (C-1 Glu1 (α,α)), 95.2 (C-1 Glu2 (α,α)), 95.1 (C-1 Glu2 (β,α)), 84.8, 82.0, 81.7, 81.5, 81.3, 79.9, 79.3, 78.8, 77.9, 77.5, 77.4, 77.2, 75.7, 75.6, 75.6, 75.5, 75.1, 75.0, 74.9, 74.8, 73.4, 72.3, 72.1, 72.0, 71.6, 71.5, 71.3, 70.4, 70.3, 70.0, 68.4, 68.1, 67.2, 67.1, 65.6, 63.8, 63.7, 34.0 ($CO\underline{C}H_2(CH_2)_5CH_3$), 31.6 ($COCH_2(\underline{C}H_2)_5CH_3$), 29.0 ($COCH_2(\underline{C}H_2)_5CH_3$), 28.9 ($COCH_2(\underline{C}H_2)_5CH_3$), 24.8($COCH_2(\underline{C}H_2)_5CH_3$), 22.6 ($COCH_2(\underline{C}H_2)_5CH_3$), 14.1 ($COCH_2(CH_2)_5\underline{C}H_3$) ppm.

(2*R*)-2-*O*-(α-D-glucopyranosyl-(1→6)-α-D-glucopyranosyl)-3-*O*-octanoyl-2,3-dihydroxypropanoic acid 2.

FT-IR (neat): 3450 (OH), 1740 and 1737 (C=O) cm^{-1}. **^{1}H NMR** (D_2O): δ 4.98 (d, *J*=3.8 Hz, H1 (α, α)), 4.96 (d, *J*=3.8 Hz, H1 (β, α)), 4.87 (d, *J*=3.6 Hz, H1 (α, α)), 4.42 – 4.36 (m, H1 (β, α), $C\underline{H}_2CH(OH)CO_2H$), 4.33 (t, *J*=4.7 Hz, $C\underline{H}_2CH(OH)CO_2H$,), 4.31 – 4.26 (m, $CH_2C\underline{H}(OH)CO_2H$,), 4.07 (d, *J*=9.6 Hz, (β, α)), 3.93 (dd, *J*=11.1, 4.2 Hz, H-6), 3.88 – 3.57 (m, H-6, H-5, H-3), 3.52 – 3.21 (m, H-2, H-4), 2.34 (t, *J* = 7.4 Hz, $COC\underline{H_2}(CH_2)_5CH_3$), 1.87 (s, $COCH_2(C\underline{H_2})_5CH_3$), 1.59 – 1.47 (m, $COCH_2(C\underline{H_2})_5CH_3$), 1.23-1.20 (m, $COCH_2(C\underline{H_2})_5CH_3$), 0.79 (t, *J* = 6.7 Hz, $COCH_2(CH_2)_5C\underline{H_3}$) ppm. **^{13}C NMR** ($D_2O$): δ 176.9 (C=O (α, α)), 176.8 (C=O (β, α)), 175.8 (C=O (β, α)), 175.7 (C=O (α, α)), 102.5 (C-1 (β, α)), 98.4 (C-1 (β, α)), 98.3 (C-1 (α, α)), 97.9 (C-1 (α, α)), 77.2 ($CH_2\underline{C}H(OH)CO_2H$ (β, α)), 77.0 ($CH_2\underline{C}H(OH)CO_2H$ (α, α)), 75.9, 75.7, 73.4, 73.1, 71.8, 71.5, 71.2, 70.8, 69.6, 69.5, 69.2, 69.0, 68.0 ((β, α)), 65.8 (C-8 (α, α)), 65.2 (C-6’ (α, α)), 60.8 ((β, α)), 60.4 (C-6 (α, α)), 33.8 ($CO\underline{C}H_2(CH_2)_5CH_3$), 30.9 ($COCH_2(\underline{C}H_2)_5CH_3$), 28.2 ($COCH_2(\underline{C}H_2)_5CH_3$), 28.0 ($COCH_2(\underline{C}H_2)_5CH_3$), 24.3 ($COCH_2(\underline{C}H_2)_5CH_3$), 21.9 ($COCH_2(\underline{C}H_2)_5CH_3$), 13.4 ($COCH_2(CH_2)_5\underline{C}H_3$) ppm.

Supplemental Figures

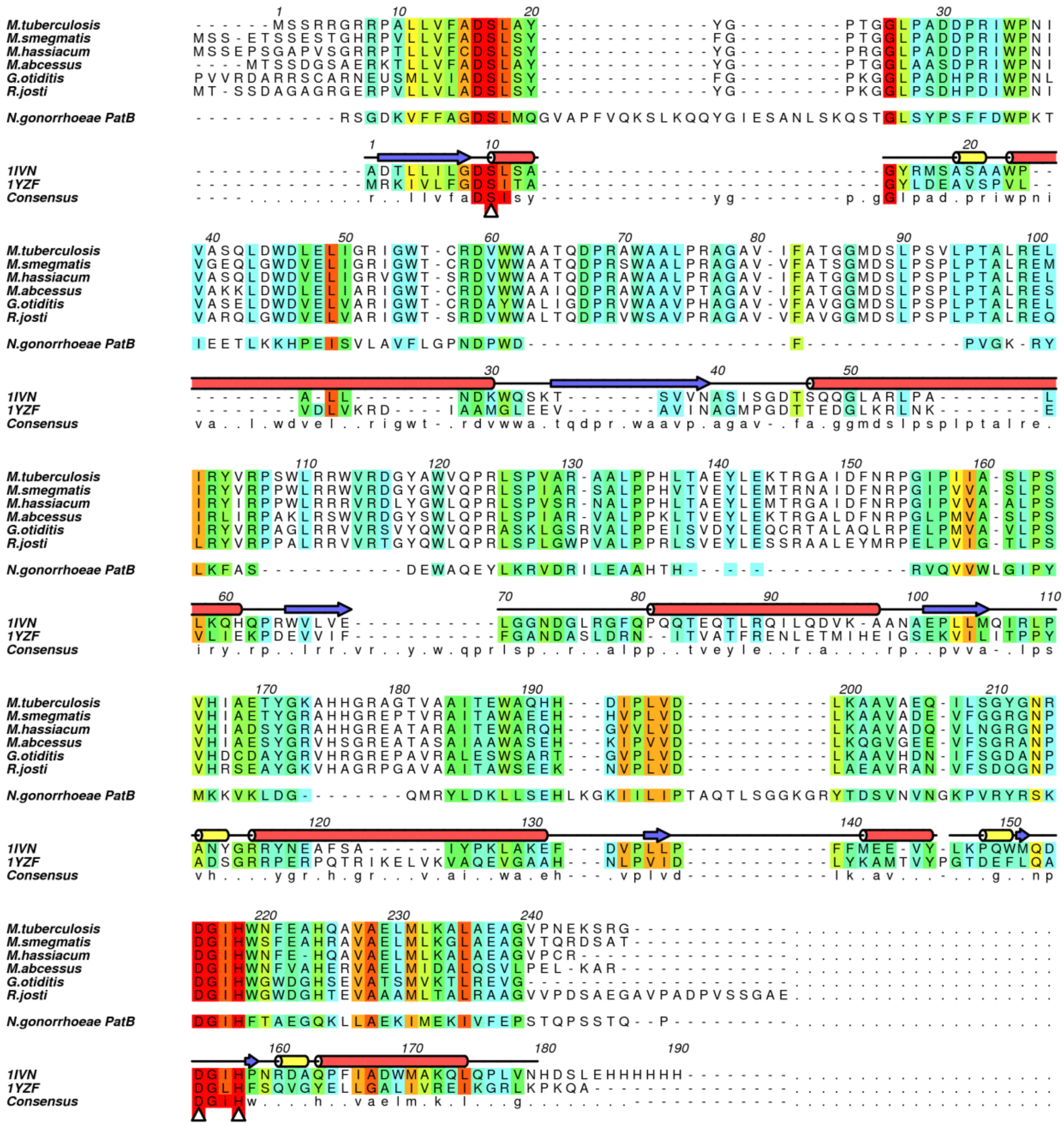

Figure S1. Amino acid sequence alignment of putative acyltransferases of the GDSL family. Selected sequences include mycobacterial octanoyltransferases from *M. tuberculosis* H37Rv (Rv2418c), *M. smegmatis* mc^{2}155 (MSMEI_4466), *M. hassiacum* (accession number EKF23139), *M. abscessus* (WP_005074557) and homologues from the closely related actinobacteria *Rhodococcus jostii* (ABG93118) and *Gordonia otitidis* (WP_007238992) as well as from PatB, the peptidoglycan *O*-Acetyltransferase B form *Neisseria gonorrhoeae*

(YP_207683) and two predicted structural homologs from *Escherichia coli* and *Enterococcus faecalis* (PDB entries 1IVN and pdb 1YZF). Strictly conserved residues are boxed in red; the conserved regions are boxed from dark orange (highly conserved) to blue (partially conserved) according to the decreasing number of conserved amino acids in the aligned column. Numbers refer to the *M. tuberculosis* OctT sequence. The secondary structure elements derived from the three-dimensional structure of predicted structural homologs are shown above the alignment (red cylinders: α-helices; yellow cylinders: 3_{10} helices; blue arrows: β-sheets). The consensus sequence was calculated for the whole sequence alignment. Possible catalytic residues are indicated by white triangles below the alignment.

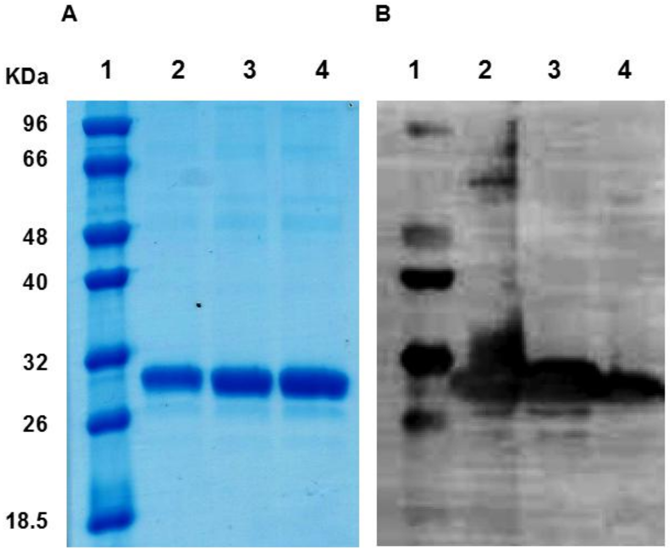

Figure S2. **Purification and oligomerization state of mycobacterial OctTs**. **A**) SDS-PAGE analysis of purified recombinant proteins. *Lane 1*, molecular weight marker; *lane 2-4*, purified recombinant OctT from *M. tuberculosis*, *M. smegmatis* and *M. hassiacum*, respectively. **B**) Western immunoblot analysis using an anti-His_6 specific antibody.

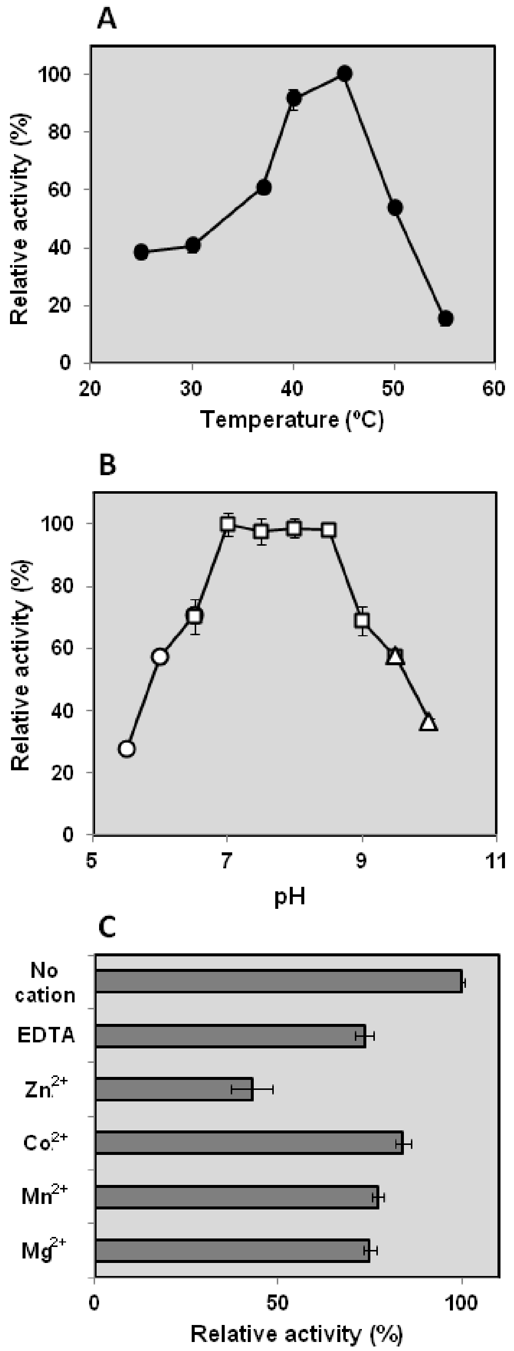

Figure S3. ***M. hassiacum* OctT properties**. **A**) temperature profile. **B**) pH dependence (circles, MES buffer; squares, BTP buffer; triangles, CAPS buffer). **C**) effect of divalent cations on activity.

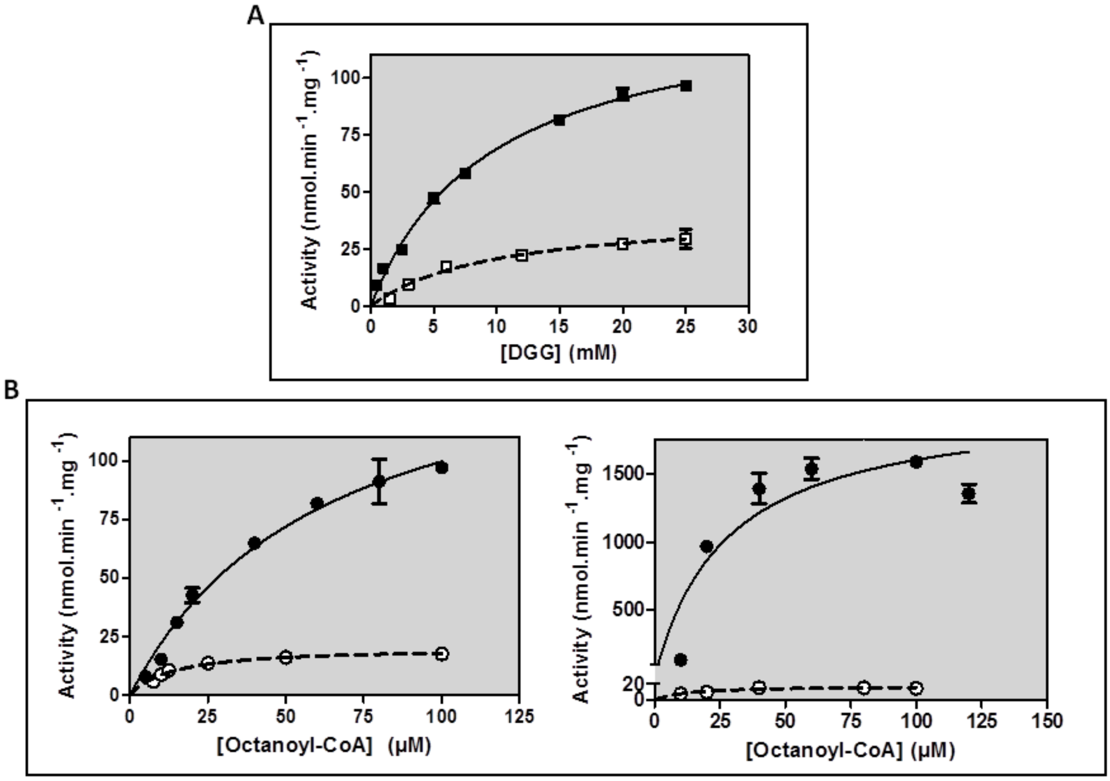

Figure S4. **Kinetic properties of recombinant OctT**. A) Plot of *M. hassiacum* OctT activity as a function of acceptor DGG concentration (Oct-CoA (filled squares) and Hex-CoA (open squares)). B) Plot of *M. hassiacum* (left panel) or *M. smegmatis* OctT (right panel) activity as a function of donor Oct-CoA concentration (DGG (filled circles) and GG (open circles).

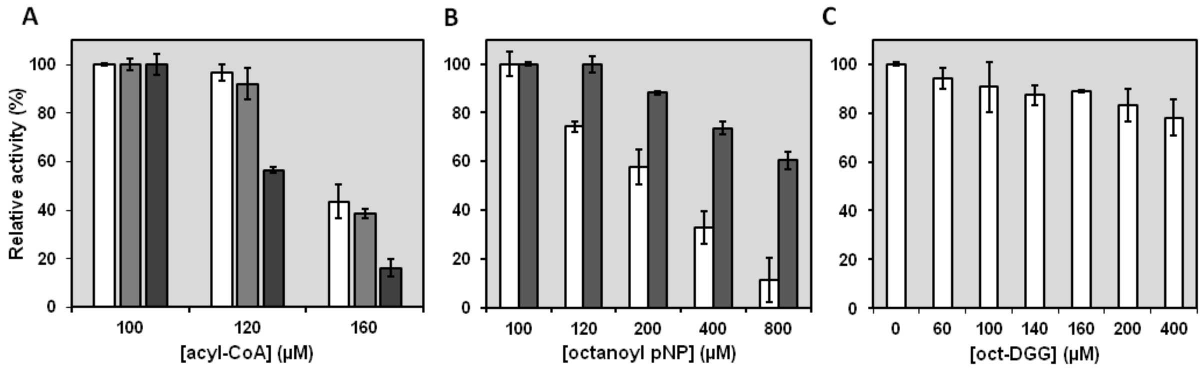

Figure S5. Recombinant OctT concentration dependent inhibition by acyl ester donor or product. **A**) Inhibition by Oct-CoA (white and grey bars) in *M. hassiacum* and *M. smegmatis*, respectively and Hex-CoA in *M. hassiacum* (black bars) in the presence of DGG (20 mM or 45 mM). B) Inhibition by Oct-*p*NP in the presence of each of the preferred glycoside acceptors GG (white bars) and DGG (grey bars) (both substrates at 20 mM). C) Inhibition by product DGG-Oct.
